# Competencies for Transformational Leadership in Public Health—An International Delphi Consensus Study

**DOI:** 10.3389/ijph.2024.1606267

**Published:** 2024-02-23

**Authors:** Barbara Maria Bürkin, Katarzyna Czabanowska, Suzanne Babich, Núria Casamitjana, Marta Vicente-Crespo, Luis Eugenio De Souza, John P. Ehrenberg, Axel Hoffmann, Rajesh Kamath, Anja Matthiä, Fredros Okumu, Elizeus Rutebemberwa, Marco Waser, Nino Kuenzli, Julia Bohlius

**Affiliations:** ^1^ Department Education and Training, Swiss Tropical and Public Health Institute, Allschwil, Switzerland; ^2^ University of Basel, Basel, Switzerland; ^3^ Department of International Health, Institute of Care and Public Health Research, Faculty of Health, Medicine and Life Sciences, Maastricht University, Maastricht, Netherlands; ^4^ Department of Health Policy Management, Institute of Public Health, Medical College, Jagiellonian University, Krakow, Poland; ^5^ Department of Community and Global Health, Richard M. Fairbanks School of Public Health, Indiana University at Indianapolis, Indianapolis, United States; ^6^ ISGlobal, Hospital Clinic—Universitat de Barcelona, Barcelona, Spain; ^7^ Research and Related Capacity Strengthening Division, African Population and Health Research Center, Nairobi, Kenya; ^8^ School of Public Health, Faculty of Health Sciences, University of the Witwatersrand, Johannesburg, South Africa; ^9^ Collective Health Institute, Federal University of Bahia, Salvador, Bahia, Brazil; ^10^ Arboretum Frutales Mayas Non-Governmental Organization Cholul, Mérida, Colima, Mexico; ^11^ Department of Health Innovation, Prasanna School of Public Health, Manipal Academy of Higher Education, Manipal, India; ^12^ Department of Environmental Health and Ecological Sciences, Ifakara Health Institute, Ifakara, Tanzania; ^13^ African Field Epidemiology Network (AFENET), Kampala, Uganda; ^14^ Department of Health Policy, Planning and Management, School of Public Health, Makerere University, Kampala, Uganda

**Keywords:** transformational leadership, competencies, competency framework, Delphi study, context

## Abstract

**Objectives:** This Delphi study intended to develop competencies for transformational leadership in public health, including behavioral descriptions (descriptors) tailored to individuals and their contexts.

**Methods:** The study involved five rounds, including online “e-Delphi” consultations and real-time online workshops with experts from diverse sectors. Relevant competencies were identified through a literature review, and experts rated, ranked, rephrased, and proposed descriptors. The study followed the Guidance on Conducting and REporting DElphi Studies (CREDES) and the COmpeteNcy FramEwoRk Development in Health Professions (CONFERD-HP) reporting guidelines.

**Results:** Our framework comprises ten competencies for transformational public health leadership (each with its descriptors) within four categories, and also describes a four-stage model for developing relevant competencies tailored to different contexts.

**Conclusion:** Educators responsible for curriculum design, particularly those aiming to align curricula with local goals, making leadership education context-specific and -sensitive, may benefit from the proposed framework. Additionally, it can help strengthen links between education and workforce sectors, address competency gaps, and potentially reduce the out-migration of graduates in the health professions.

## Introduction

Leadership in a complex and interconnected world is particularly successful if it is adaptable and open to change. Leaders might benefit from pursuing a transformational approach [1, 2]. In the aftermath of COVID-19, contemporary environmental, social and technological trends necessitate transformational leaders adept at handling critical ethical issues. This includes managing resource allocation implications in public health crises, balancing individual privacy rights with public health surveillance, addressing health disparities in underserved communities or navigating the ethical incorporation of emerging technologies, like artificial intelligence, into public health decision-making.

Transformational leaders can emerge at any level [3], in any context, position or sector [2]. They take a whole-system view, deal well with uncertainty, stimulate reflection, and guide their teams to shape the future [4, 5]. Their individual leadership qualities are supported by context-dependent qualities of different world regions, expanding the scope to a global dimension [6, 7]. Transformational leaders in public health may emerge naturally, but it should also be possible to train them with different educational approaches that focus on acquiring competency-specific behaviors to improve public health leadership across a wide range of positions, environments, and contexts [8–10].

Existing competency frameworks categorize “leadership” into a separate domain and describe its behaviors [8, 11–17], but most do not explicitly cultivate transformational leadership competencies and none acknowledge that optimal leadership must be developed in context. Only Kouzes and Posner’s framework can be said to be oriented to transformational leadership [15]. However, its scope is limited to organizational development without providing clear methodology [15]. We need a competency framework to develop transformational leaders in public health, leaders capable of adapting to the changing demands of a rapidly evolving world [18]. The Delphi technique is a common and comprehensive technique to systematically define competencies [8, 11, 12, 14, 16, 19–25] (Box 1) by leveraging and reflecting the collective insights of diverse experts, allowing for anonymous input to foster consensus, and iteratively refining ideas. We did a Delphi study to develop and define competencies for transformational leadership in public health and to generate behavioral descriptions (descriptors) for individual, context-specific and context-sensitive competencies.

## Methods

### Planning and Design

**FIGURE 1:**
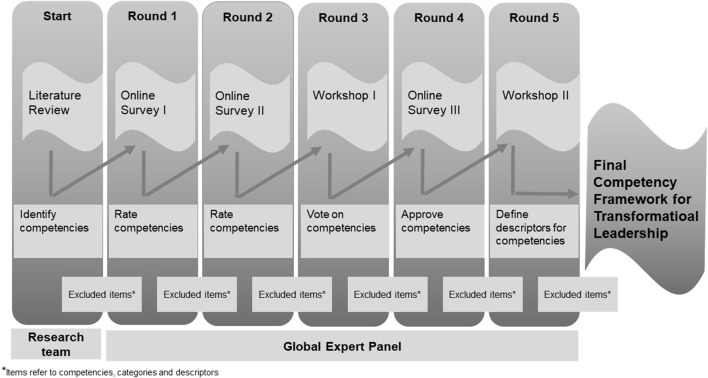
Delphi process. Competencies for transformational leadership in public health—an international Delphi consensus study (Allschwil, Switzerland, 2023).

The Delphi process consisted of five rounds (Figure 1), including three asynchronous online “e-Delphi” consultations and two real-time online workshops with a selected group of experts. Our study report follows Guidance on Conducting and REporting DElphi Studies (CREDES) [26], adheres to The COmpeteNcy FramEwoRk Development in Health Professions (CONFERD-HP) [27] and follows internationally accepted recommendations [28–31] (Figure 1). We identified relevant competencies through literature review and invited experts to evaluate, rank and suggest changes. We defined consensus through a stepwise process. We statistically analyzed expert responses from the first two Delphi Rounds, supplemented by qualitative content analysis. In Delphi Round 3, we also conducted a consensus vote. We analyzed video recordings and group notes to track the process and deal with non-consensus and divergent voting results. After each round, the research team determined saturation and group consensus.

### Study Setting

We collected data from an international group of experts from 30 countries in low and middle income countries as well as high income countries, using the web-based platform EvaSys [32]. To reduce bias, we conducted anonymous online survey rounds with selected experts combined with two real-time workshops via the Zoom videoconferencing software [33], after which experts used a chat-based application to anonymously rank statements via voting [32].

### Literature Review

To date, there was no consensus-based competency framework for transformational leadership. We based our study on the WHO-ASPHER Competency Framework [11] and other referenced frameworks [8, 12, 14, 16, 19–22, 24, 25]. We chose these frameworks for their approach to consensus-based competency development. Additionally, we selected them for explicitly defining domains or competencies in leadership and management. We singled out relevant framework references listed in the WHO-ASPHER Competency Framework and tabulated them, then categorized the key domains and competencies in the field of management and leadership. To ensure the competency framework to be innovative, applicable in practice and most importantly transformative, we considered transformation-oriented theories such as Theory U [2] in the consensus-based development of the descriptors (Supplementary Box S9).

### Composition of Expert Panel

Members of our research team and network suggested and nominated transformational leaders from their professional and scientific trajectories as experts for this study. We specifically asked our research partner Consortium for Advanced Research Training in Africa for nominations. Our sampling frame included global experts in public health. Although this approach only allowed for partial representativeness, and in our case skewed towards African experts, we aimed to gather a diverse pool of expertise (Supplementary Table S3) [26]. Following Van Loo and Semeijn’s (2004) methods to defining and measuring competences, we included experts from different perspectives or relevant research fields for competency development, e.g., education, labor, human resources, or organization, research and development and policy [10]. Following Van Loo and Semeijn’s (2004) approach, we included experts from different perspectives or relevant fields of research for competency development, e.g., education, work, human resources or organization, research and development and policy [10].

These practices ensured that our resulting list of competencies would be based on reality and need, increasing the likelihood that graduates would be able to work across sectors [14]. Our focus on diversity of gender, age, professional background and geography within experts and in contextual considerations ensured our framework was broadly applicable. Public health was not explicitly offered as a perspective. Rather, we assumed that all experts, selected by snowball sampling, worked in public health or related research fields (Supplementary Table S3).

A formal letter and video invitation was sent to 87 people. Willingness to participate in subsequent Delphi rounds (online surveys and online workshops) was a prerequisite for participation. After each session of the online survey, we reminded experts who had not responded. Of those invited, 60 agreed to participate (69%). Median age of experts in Delphi Round 1 was 46–56 years; 59% (*n* = 26) were women (men: *n* = 18, 41%). Among the 44 experts in Delphi Round 1, 33% were from Europe, 29% from Africa, 21% from North-Central or South America, and 14% from Asia. Of the 43 people who indicated their workplace, 35% were from Africa, 30% from Europe, 21% from North-Central or South America, and 14% from Asia. Perspectives could overlap, and so 55% (*n* = 24) of experts offered an “Education” perspective, 52% (*n* = 23) a “Research and Development” perspective, 21% (*n* = 9) a “Policy” perspective, and 12% (*n* = 5) a “Human Resource or organizational” perspective.

### Delphi Process

#### Developing the Survey Instruments

We used an iterative process to develop the questionnaire for Delphi Round 1 and launched it on the EvaSys software platform with the support of a specialist. The research team, a co-researcher, and three external laypersons reviewed and piloted the questionnaire, whereupon the research team approved the final version in two consultation sessions [26]. The questionnaire sought expert information, study details and information on transformational leadership competencies. Central in our approach was asking experts to rate competencies we had compiled from the previous literature review of competency frameworks (Supplementary Figures S3–S6).

#### Delphi Rounds 1 and 2 Online Survey

Experts ranked their agreement on the importance of the Competencies for Transformational Leadership on a 7-point Likert scale (1 = not important, 2 = low, 3 = slightly, 4 = neutral, 5 = moderately, 6 = very, 7 = extremely important), and also scored the individual work context in which they considered these competencies valuable (these latter data were not part of this study).

Experts could suggest alternative wording and add competencies, make general comments about competencies and comment on their categorization in “Knowing,” “Doing” and “Being” in Delphi Round 2 [34]. We circulated newsletters after the online surveys, reporting clustered recurring themes and arguments (called ‘Golden Nuggets’). These newsletters ensured quality, transparency, clarity and plausibility by explaining our decisions to adjust competencies and categories (Supplementary Boxs S6, S7).

#### Delphi Round 3—Consensus Workshop

We organized an online Zoom workshop and randomly formed diverse groups of experts. We aimed to gather different perspectives and discuss the classification of competencies into categories derived from the qualitative analysis of the previous two rounds. We appointed experts from our collaborative scientific community 1 week before the workshop to act as group moderators (facilitating the discussion in small groups) or rapporteurs (documenting and presenting the results of the discussion in plenary). The workshop started with a questionnaire asking all experts to anonymously categorize the revised list of competencies. Results were then used to discuss how to categorize the competencies assigned to each group. To ensure a comprehensive consideration of all competencies and to avoid compartmentalization within groups, we assigned each competency twice. All groups then discussed their findings in plenary, allowing all experts to actively consolidate and build consensus (Supplementary Figures S1, S2).

#### Delphi Round 4—Online Survey

We transferred the fourth version of the consolidated competency list to an anonymous, voluntary survey. We invited all experts to approve the final list and comment (optionally) (Tables 1, 2).

**TABLE 1:** Evolution path of final competency lists version 1 & 2 for transformational leadership over five rounds of adjusted e-Delphi. Competencies for transformational leadership in public health—an international Delphi consensus study (Allschwil, Switzerland, 2023).

Delphi round 1 (*n* = 44, RR = 73%) competency list version 1^a^ (CL1)	R1^b^	Reference from literature	Delphi round 2 (*n* = 38, RR = 63%) competency list version 2^a^ (CL2)	R2^b^	Ref. CL1
C02_TL Is able to discern interdependences and power relationships within and outside the organisation (including formal rules and structures, decision-making processes, and influencers) and addresses barriers to successful collaboration to improve public health services	97.7	73, 76, 67, 101	C10_TL Builds trust and inspires others to commit to a common goal	97.4	C14
C10_TL Facilitates the development of others as leaders and teams for implementing health initiative	97.6	10, 39	C11_TL Empowers others through an honest, respectful, and sensitive way to fully capitalise collective wisdom and to synergise competencies for public health	97.4	C23
C11_TL Fosters an environment including professional development opportunities that encourages professional and personal growth and the transfer of knowledge to future talent	95.4	18, 41	C01_TL Demonstrates adaptability and flexibility in collaborating and achieving shared goals	94.7	C17
C34_TL Acts according to ethical standards and norms with integrity including professional accountability, social responsibility and the public good	95.3	7	C08_TL Demonstrates perseverance, optimism, and resilience in uncertain and challenging times”	94.7	C28
C14_TL Inspires, motivates, builds trust, and guides others to engage and to work towards a shared vision, programme and/or organisational goal	93.1	11, 21, 36, 19	C03_TL Cultivates professional and personal growth of team members, internal and external stakeholders based on identified development needs	94.6	C9, C10, C11
C26_TL Recognises one’s emotions and is aware of how one’s own beliefs, values and behaviors affect one’s own decision-making and the reactions of other	93.1	43, 44	C14_TL Acts ethically and is paragon of integrity, fairness, and transparency	92.2	C34
C23_TL Motivates others in an honest, respectful, and sensitive manner to achieve high standards of performance and accountability	93	23, 24, 40	C12_TL Creates synergies and fosters a collaborative environment through trust and participatory decision making	91.9	C16
C27_TL Critically reviews and evaluates own practices in relation to public health principles, including critical self-reflection	90.7	45	C13_TL Builds coalitions with diverse stakeholders and co-creates innovative solutions and health interventions in alignment with the organization’s mission and values	91.8	C21
C09_TL Senses development needs and supports roles, abilities, and responsibilities of others, including external stakeholders	90.7	8, 9	C02_TL Chooses participatory leadership practices and systems-thinking techniques to nourish diversity and inclusion in interdisciplinary and intersectoral collaborations	86.5	C03, C13, C37
C16_TL Creates group synergy in pursuing collective, interdependent goals, common values, and norms to foster a collaborative environment including respectful communication and participative decision-making	90.7	96, 34, 35	C06_TL Recognises own emotions and engages in awareness-based practices for self-care and self-reflection of own work	81.6	C26, C27
C17_TL Demonstrates practicality, flexibility, and adaptability in the process of working with others, emphasizing achieving goals as opposed to rigidly adhering to traditional and commonly used work method	88.4	37	C04_TL Adapts flexibly to a variety of situations, individuals, and groups	78.3	C30
C30_TL Appreciates diverse perspectives on an issue and flexibly adapts to a variety of situations, individuals, or groups	88.1	48, 50, 94	C09_TL Strives to challenge interdependencies and power relations within and outside the organization and addresses social inequalities and obstacles with an activist and entrepreneurial spirit	75	C02, C36
C37_TL Demonstrates cultural awareness and sensitivity in communication with diverse populations including the understanding of unspoken, partly expressed thoughts, feelings, and concern	86.1	37	C05_TL Strives to uncover patterns and complex relationships in a variety of situations and contexts”	65.7	C01
C21_TLCommunicates the organisation’s mission and values to stakeholders and effectively shares information and responsibility at different organisational levels to gain political commitment and social acceptance	86.1	28, 29	C15_TL Is able to act on the wicked complexity of multifaceted systems	65.7	Suggestion KC
C29_TL Self-regulates disturbing emotions and impulses and restrains negative actions when faced with uncertainty, work-related stress or opposition and hostility from other	86.1	46, 49, 99	C07_TL Applies self-regulation techniques and operates out of vulnerability	62.9	C29
C28_TL Demonstrates persistence, optimism, perseverance, resilience, and the ability to call upon personal resources and energy when delivering tasks within a limited period or at times of challenge	86	51, 58, 59			
C36_TL Senses power relationships and social inequalities and takes an active interest in others’ feelings, perspectives, or emotional currents	86	1, 2, 60			
C13_TL Effectively manages people, specifically by providing clarity on task responsibility, ensuring sufficient resources and training and provides regular feedback on performance	86	20			
C01_TL Is able to identify patterns and underlying issues across situations and in seemingly random items	86	91, 72			
C03_TL Applies principles of systems thinking within systematic enquiry to manage relationships with stakeholders in interdisciplinary and intersectoral projects and programmes	85.3	74, 75, 79			
C32_TL Is willing to pursue lifelong learning including self-assessing and addressing own development needs based on career goals and required competencies	83.7	47, 54			
C12_TL Develops capacity, including strategies at the individual, organisational and community level for ongoing change and self-renewal	83.5	16, 17			
C31_TL Strives to meet a standard of excellence through proactiveness, innovativeness, risk-taking and by acting on evidence-based professional practice	83.4	52, 55, 57			
C04_TL Guides organisational decision-making and planning in relation to strategic goals, based on internal performance evaluation and external environmental research	81.4	63, 64, 66			
C24_TL Effectively works in professional networks and partnerships across sectors to generate evidence and to implement programmes and services based on common goals and priorities	81.4	25, 26, 27			
C18_TL Effectively leads interdisciplinary and diverse teams by negotiating and resolving disagreements to work in a coordinated manner in various areas of public health practice	79.1	33, 38, 4			
C15_TL Creates change strategies (behavioral and/or cultural) and catalyses the emerging mind-set that integrates people, communities, processes, and content needs, to support new business directions	79.1	6, 22, 32, 71			
C38_TL Appraises the needs and concerns of internal/external stakeholders (e.g., committees, working groups, country representatives, etc.) to derive sound recommendations and/or solutions from this	79.1	97			
C25_TL Applies effective techniques for generating win-win outcomes with people who might be important for achieving strategic-related goals	78.6	42, 100, 78			
C35_TL Manages conflict-of-interest situations as defined by organisational regulations, policies, and procedures	74.4	56			
C08_TL Continuously generates and communicates (new) information respectfully and effectively through a range of modern media channels to lay, professional, academic, and political audiences	74.4	—			
C33_TL Assumes responsibility for one’s interventions by recognising opportunities and acting efficiently at the appropriate moment and within the given deadline	74.4	92			
C22_TLCommunicates health messages, facts, and evidence effectively and strategically by defining the target audience, listening, and developing audience-appropriate messaging and within the context of translating science and evidence into practice	66.7	30, 5			
C05_TL Uses conscious process thinking to design and implement strategic planning processes aligned with regulatory and statutory requirements and integrated with all interdependent system	65, 8	70, 65, 68, 69, 65			
C06_TL Applies principles of human, financial, project and operational resource management including risk assessment and quality improvement methods to efficiently organise project workflows and to improve organisational performance	65, 2	15, 80, 81, 82, 83, 84, 86, 87, 88, 95			
C07_TL Deploys (digital) technologies, good practices, and social media to implement information systems and to manage, analyse and store data and health information	59.5	(85, 89, 90)			
C20_TL Facilitates communication within and between organisations by delivering outputs such as meeting agendas, presentations, reports, and project dissemination	52.4	14			
C19_TL Effectively plans the allocation of work tasks including the development of job descriptions, interviewing and selecting candidates to achieve the goals set by the organisation	52.4	12, 13			

^a^ Explanation for reformulation/adaptation of competencies to be found in Supplementary Material: *Analysis of expert feedback*.

^b^ Rating on the importance of competencies for transformational leadership descending by the percentage of consent during Delphi round 1 and 2, level of agreement, sum value scale 6 + 7.

**TABLE 2:** Evolution path of final competency lists version 3–5 for transformational leadership over five rounds of adjusted e-Delphi. Competencies for transformational leadership in public health—an international Delphi consensus study (Allschwil, Switzerland, 2023).

Delphi round 3 (*n* = 40, RR = 66%) competency list version 3 (CL3)^a^	Ref. CL 2	Delphi round 4 (*n* = 11, RR = 6%) competency list version 4 (CL4)^a^	Level of agreement Yes/No	Ref. CL3	Delphi round 5 (*n* = 25, RR = 41%) competency list version 5 (final)^a^	Ref. CL4
C01. Co-creates value based, innovative solutions	C13	C01. Adapts to the needs of the eco-system to achieve shared goals	70	C02	C01 Adapts according to the needs of the eco-system	=C01
C02. Adapts appropriately to the needs of the eco-system to achieve shared goals	C01, C04	C02. Inspires others to commit to a common goal	90	C04	C02 Inspires others to commit to common goals	C02
C03. Initiates, monitors and measures coalitions and partnerships with diverse stakeholders	C13	C03. Empowers others to build upon their competencies and wisdom	100	C05	C03 Empowers others to fully capture and build upon their competencies and wisdom	C03
C04. Inspires others to commit to a common goal	C10	C04. Practices participatory and inclusive leadership	100	C09	C04 Practices participatory and inclusive leadership	C04
C05. Empowers others to fully capture and build upon their competencies and wisdom	C03C11	C05. Creates synergies and fosters a diverse environment	90	C06	C05 Creates synergies and fosters a collaborative environment	C05
C06. Creates synergies and fosters a collaborative environment	C12	C06. Initiates and monitors coalitions and partnerships with diverse stakeholders	90	C03	C06 Proactively manages partnerships with diverse actors	C06
C07. Personifies optimism and perseverance to build resilience in uncertain and challenging times	C08	C07. Personifies optimism and perseverance to build resilience in challenging times	80	C07	C07 Personifies optimism to build resilience and perseverance in challenging times	C07
C08. Acts ethically, always has integrity and is perceived to be always fair	C14	C08. Acts ethically, with integrity and is perceived to be always fair	90	C08, C12	C08 Acts with integrity	C08
C09. Practices participatory leadership	C02	C09. Acts in complex and multifaceted systems	90	C10	C09 Operates effectively within and across complex and multifaceted systems	C09
C10. Acts in the complexity of multifaceted systems	C02, C05, C15	C10. Challenges the unequal distribution of power in internal and external relations	77.8	C13	C10 Pursues strategic approaches for value-based, innovative solutions	C11, C12
C11. Nourishes inclusion and diversity to advance interdisciplinary and intersectoral collaboration	C02	C11. Co-creates value based, innovative solutions	80	C01		
C12. Applies self-regulation techniques to operate authentically	C06, C07	C12. Adopts an entrepreneurial approach in finding future oriented solutions	70	C14		
C13. Challenges power relations internally and externally to reduce inequality	C09					
C14. Adopts an entrepreneurial approach in finding future oriented solutions	C09					

^a^ Explanation for reformulation/adaptation of competencies to be found in Supplementary Material: *Analysis of expert feedback*.

Box 1 **Relevant competency-based frameworks in public health (Allschwil, Switzerland, 2023).**

• The WHO-ASPHER Competency Framework for the Public Health Workforce in the European Region [11] combined literature review, several rounds of expert and stakeholder exchanges, and a consensus survey. Leadership and systems thinking, comprise one of its ten domains. Transformational leadership is not a key focus.
• The Regional Core Competency Framework for Public Health (RCCFPH) for the Americas [12] was developed under the guidance of a Regional Steering Group and six Expert Committees within three Regional Workshops and an external meeting. Leadership is considered an “attribute” and a cross-cutting dimension within each domain.
• The Doctor of Public Health (DrPH) Core Competency Model [13] was developed in a modified Delphi process comprising three Delphi rounds and additional conference calls, followed by participation of an advisory panel and seven working groups on individual competency domains to illustrate domains and competencies in a uniform model. Generalist leadership management and research skills were emphasized.
• The National Aboriginal and Torres Strait Islander Public Health Curriculum Framework [14] was developed after consulting the national network of Indigenous academics, practitioners, policymakers, and after holding special learning forum and receiving feedback from Indigenous students. The six public health core competencies do not include leadership.
• The Leadership Practices Inventory [15] one of the most widely used inventories, consolidated thousands of stories, each an answer to the question of what the leader does when they perform their best. The inventory focuses on leadership behavior within five practices “Model the Way,” “Inspire a Shared Vision,” “Challenge the Process,” “Enable Others to Act,” and “Encourage the Heart.”
• The Core Competencies for Public Health Professionals [16] were the product of steady revision and review over many years to ensure their relevance and timeliness. Leadership and systems thinking skills made up one of eight domains that described skill areas within public health.

#### Delphi Round 5—Operationalization Workshop

We developed a stage model (see Figure 3) to reflect the gradual acquisition of skills. We drew on the Dreyfus scale [35] which we adapted: we defined four stages of transformational leadership instead of the five levels described by Dreyfus. We discarded the “novice” and “advanced beginner” stages as irrelevant and started with “competent” as the first stage. The latter stages were not formally described because both contextual understanding (novice) and emotional closeness should be present for the development of transformational leadership competencies.

We added the “transformational leadership stage”—the highest achievable level [35, 36]. We extracted appropriate examples of behavior (descriptors) from the competency frameworks we used to create our initial list of competencies (version 1) as well as from the feedback on our suggested reformulations from the online questionnaire rounds. Finally, we synthesized and thematically organized the descriptors assigned to the ten competencies [11, 12, 15–17, 37].

We held a second online Zoom workshop to harmonize competency descriptors. To prepare, experts elaborated descriptors for their assigned competencies. During the workshop, the groups each discussed descriptors for two competencies and their allocation to the stage model of competency development (Tables 1, 2).

### Data Analysis

For Delphi Round 1, we set a threshold of 85% agreement for each competency to determine the inclusion or exclusion of competencies [38]. We chose this conservative threshold because of the large number of initial competencies. Experts could suggest additional competencies and increase the number for Delphi Round 2. The 85% threshold required experts to score 6 or 7 for a competency on a 7-point Likert scale. For Delphi Round 2, we lowered the threshold to 80% for the following reasons: 1) competencies scored high in Delphi Round 2, 2) the number of competencies dropped from 38 in Delphi Round 1 to 15 in Delphi Round 2 and 3) we needed to provide sufficient basis for discussion in Delphi Round 3. We sorted the competencies according to level of agreement, color-coded corresponding passages and identified synergies between the competencies that achieved 85% (Delphi Round 1) or 80% (Delphi Round 2). We then reordered competencies by descending agreement scores, analyzed and selected relevant text passages from open-ended responses (competencies, categories, comments, and recommendations). We clustered responses by content and extracted alternative formulations for competencies. We grouped similar comments and recommendations into Golden Nuggets, supported with text examples. To systematically assess written comments we applied conventional (using coding categories) and summative (counting and comparisons) qualitative content analysis [39]. We counted how often certain terms appeared in selected segments. We then created a coding scheme consisting of categories, subcategories, and describing indicators and definitions. We reviewed and piloted the coding scheme within the response texts and conducted a conventional analysis by coding with the real text [26]. We consolidated Likert scale ratings of all competencies and revised the competency list after Delphi Rounds 1 and 2. We determined means, median and mode, standard deviations, and inter-quartile ranges to describe aggregated ratings. We synthesized the workshop data (online survey, consensus vote, minutes, and video recordings) into a word table (Delphi Round 3). We jointly discussed and evaluated the plausibility and relevance of feedback from Delphi Round 4 and developed version 5 of the competency list. We analyzed data from the operationalization workshop (Delphi Round 5) to define the most suitable descriptors for each competency. Here we transcribed video recordings and used them together with group protocols to adapt and complement descriptors. All descriptors were critically reviewed by a specialist for gender and diversity aspects [26] (Tables 1, 2). Additional information: We analyzed Delphi Round 1 and 2 data similarly. We used the same coding scheme to analyze free text. During each Delphi round, experts and the team refined the Competency Framework for Transformational Leadership and adjusted the competency list (Supplementary Figures S1, S2).

### Ethical Considerations

We received an ethics waiver from the Ethics Committee Northwest and Central Switzerland (Req-2020-01425). Experts were informed of the aims, purpose, procedures, potential risks, and benefits of the study. We explained that the study was voluntary and that they could withdraw at any time without consequence.

No personal identifiers or names were used in the analysis or in the research reports. During the real-time consensus workshop, particular attention was paid to confidentiality by anonymizing the thematic inputs.

## Results

### Literature Review

Our research team analyzed the WHO-ASPHER Competency Framework [11] and related frameworks [8, 12–25], and extracted and reviewed a draft list of 100 competencies for transformational leadership (Supplementary Table S1). We chose the subordinate categories “Knowing,” “Being” and “Doing” and distinguished between “Educational Context,” and “Transition” and “Professional Context” [34, 37] to indicate transformational leadership resulted from the process by which individuals developed competencies. We chose category-related clusters and adjusted thematic overlaps by merging and consolidating competencies until there were only 38 which we transferred into a draft questionnaire (Supplementary Box S1).

### Delphi Procedure Results

**FIGURE 2:**
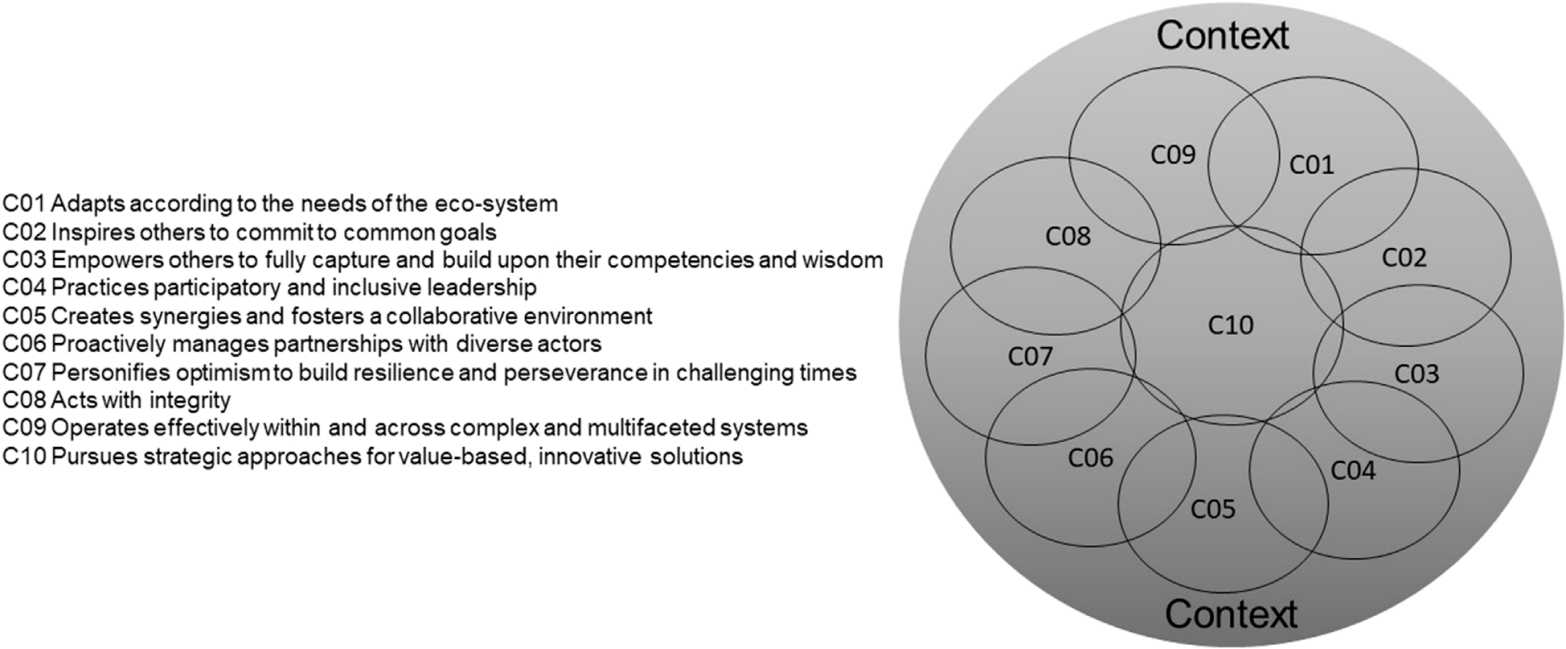
Final competency framework. Venn diagram demonstrating individual competencies as interrelated, overlapping and context-dependent. Competencies for transformational leadership in public health—an international Delphi consensus study (Allschwil, Switzerland, 2023).

**FIGURE 3:**
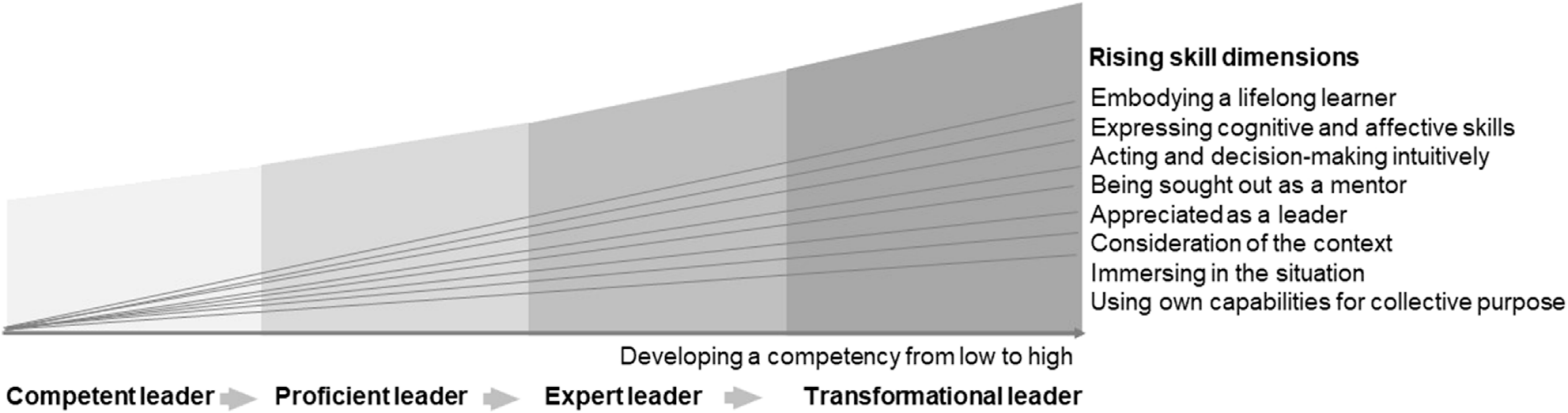
Developmental stages of competency development. Competencies for transformational leadership in public health—an international Delphi consensus study (Allschwil, Switzerland, 2023).

In five rounds of adjusted e-Delphi, we condensed the initial 38 competencies into 10 competencies and 4 categories constituting the final Transformational Leadership Competency Framework (Figure 2). This development path includes versions 1 to 5. We identified four competency stages (Figure 3) and systematically developed corresponding descriptors as part of a self-assessment tool (Supplementary File—Self-assessment Tool—Competencies for Transformational Leadership). Tables 1, 2 show the ratings and the evolution path of the final competencies, including list version 1–5 for all rounds. We described our results in the order we obtained them below.

Of the 38 competencies in Delphi Round 1, over 85% of experts agreed on 20 competencies. The categories of “Knowing,” “Being,” and “Doing” were discarded after Delphi Round 1 since experts concluded that it was not appropriate to assign competencies to these categories. After analyzing the feedback from Delphi Round 1, we reduced the number of potentially relevant competencies to 15 and generated the following new thematic categories: “Process,” “Context,” “Self,” “Systems Thinking,” “Relationships,” and “Mind-set.” Our qualitative analysis of experts’ written responses in Delphi Round 2 confirmed the appropriateness of these categories (Supplementary Figures S1, S2). Newsletter 1 (after Delphi Round 1) and Newsletter 2 (after Delphi Round 2) synthesized the qualitative analysis with illustrative examples. Of 15 competencies in Delphi Round 2, over 80% of experts agreed on 10 competencies. The broad agreement of the group led to the conclusion that saturation had been reached, meaning that no further competencies should be added. Our analysis of the Likert-scale ratings and the qualitative data analysis of open-ended feedback by the research team resulted in an adapted list of 14 competencies after Delphi Round 2.

In Delphi Round 3 (Consensus workshop), we then asked groups to assign single competencies to the before mentioned categories. Results from the workshop were as follows: 1) all competencies are context-specific and -sensitive, thus experts no longer consider “Context” as a single category; 2) context forms a superordinate thematic circle and precedes all other categories to reflect the complexity of different working contexts; 3) context determines the level to which the performance of competencies can be realized; 4) categories cannot be made distinct, since they are interrelated and merge (gradient); 5) the categories “Self” and “Mind-set” should be combined. After Delphi Round 3, we reduced the competency list from 14 to 12 and adjusted the competency framework (Figure 2) for the Context superordinate for three reasons: 1) to make clear that context determines the level at which a competency can be performed; 2) to illustrate that the relevance of a competency depends on its context; and 3) to highlight the possibility single competencies can be proportionally assigned (weighted by context) to the four categories of “Process,” “Self/Mind-set,” “Systems Thinking,” and “Relationships.”

Delphi Round 4 closed the process of developing the competency list with a voluntary and anonymous EvaSys survey round. Two of the 12 remaining competencies after the Consensus workshop received 100% agreement. The research team assessed agreement on the competencies and the open-ended comments and arrived at 10 final competencies (Supplementary Box S8). In Delphi Round 5 we assigned literature-based behavioral examples (descriptors) to each of the 10 final competencies [2, 11–13, 15–17, 37] (Supplementary Box S9). In parallel, we used the Dreyfus scale as an example when we developed the Developmental stages of competency development (Figure 3) to classify descriptors [35, 36]. We asked experts to identify descriptors for the ten core competencies of transformational leadership and assign them to the stage *Competent leader* (Stage 1), *Proficient leader* (Stage 2), *Expert leader* (Stage 3), and *Transformational leader* (Stage 4). Experts in the operationalization workshop agreed that they could not assign descriptors to the stages model because stages are not distinct. Instead, they agreed it would be best to take a cumulative approach to assessing degrees of transformational leadership by using the number of transformative behaviors an individual exhibits to quantify their level of competency attainment. We thus drafted a self-assessment tool to estimate the level of competency reached: Users rate themselves on a scale for each descriptor and are scored by cumulative points. We will validate this tool in a follow-up project once we define the numerical ranges that will help us determine how to assign the user to the appropriate stage (Figure 3).

## Discussion

Our international Delphi consensus study generated the first set of transformational leadership competencies and behavioral descriptors for public health. This unique framework integrates context as a key factor in developing a fluid, integrated concept of transformational leadership competencies.

### Strengths and Limitations of the Study

While experts came from 30 countries, engagement must be regarded as partial and incomplete, especially as there was an overrepresentation of African experts. This limitation might restrict the applicability and robustness of the competencies due to the absence of perspectives from broader and more diverse contexts. Some experts who attended the first workshop did not return for the second. It is possible our results are skewed towards the views of those experts who were most interested in the study. Real-world testing of the competency framework will show whether the competencies are indeed context-specific and context-sensitive. Future researchers could seek mandatory consent to participate in all rounds of Delphi in advance to avoid this problem. Our study was strengthened by the mix of synchronous and asynchronous phases: the asynchronous phases offered experts a high degree of anonymity, while the synchronous phases allowed experts to discuss and take positions within the group.

We singled out the work of Kouzes and Posner [15] and the WHO-ASPHER Framework [11] because of the thematic relevance of their descriptors and their classification into levels. The five overarching practices (Leadership Practices Inventory) Kouzes and Posner describe were key to the development of our competency framework, and particularly our descriptions of practices [15]. However, the Leadership Practices Inventory [15] does not include transparent, open-access representations of their methodological approach to developing the practices they described and their accompanying behaviors. Our study aligns with the WHO-ASPHER Competency Framework [11], the DrPH Core Competency model [8, 13], the Leadership Practices Inventory [15] and the Core Competencies for Public Health Professionals [16] in their approach to define behavioral descriptions for competencies. The WHO-ASPHER framework provides level descriptors for each levels (competent, proficient, expert), but we decided to take a cumulative approach to guide our assessment of the degree to which competencies are achieved and mastered. Our experts agreed, after the second Delphi workshop (Delphi Round 5), that the stages would overlap too much to be clearly divided into “Competent leader,” “Proficient leader,” “Expert leader” and “Transformational leader” (see Figure 3).

Most competency frameworks assume the sequence of competencies is static and usually do not acknowledge the influence of context on the expression of individual competencies. In contrast, our study did not assume that a fixed list of competencies would be universally relevant and globally applicable, so we integrated context, using it as a starting point to determine the relevance of competencies and to direct the acquisition/development of competencies. Though the Australian Framework does explicitly address local context (Aboriginal and Torres Strait Islander health in urban, rural and remote contexts) [14], as does the Doctor of Public Health (DrPH) Core Competency Model [8, 13], which emphasizes the national context and is contextually anchored in research practice-relevant skills, we exceeded their scope by including a high degree of global contextualization, facilitated by our multinational and multiperspective panel of experts from 30 countries. Our framework is complementary to existing competency frameworks in public health that address core public health tasks such as surveillance and monitoring because we claim that transformational leadership is not confined to a single domain within a defined category (see the category “Relations and Interactions” in the WHO-ASPHER Framework) [11] but rather a dimension that cross-cuts domains (see Regional Core Competency Framework for Public Health (RCCFPH) [12]). Drawing on this transformational leadership may lead to more unisonous execution of core public health tasks.

### Significance of the Study: Possible Mechanisms and Practical Implications

Transformational leadership competencies matter in the specific situations where they are applied. This makes them globally valuable and relevant across different work cultures. We anticipate that our framework will be of interest to educators who are responsible for curriculum design, and especially those who want to align curricula with local goals and contextualize leadership education. In practice, the framework and its accompanying descriptors should guide the selection and alignment of curricular elements such as learning objectives, methods, activities and assessments. Specifically, this involves examining curricula to assess whether they teach the skills necessary for students to develop certain competencies. This comparison includes evaluating the content and methods used in educational programs (such as textbooks or lesson plans) against a list of skills (descriptors) required for a specific task or job (Supplementary File–Self-assessment Tool: Competencies for Transformational Leadership).

Context sensitivity and specificity is of overriding relevance, especially in countries where educators seek to stem the out-migration of graduates in the health professions [1, 6, 7, 40, 41]. To successfully develop transformational leaders, educators must teach students how to adapt their current knowledge to use in a new ecosystem [18, 42–44]. Our 10 competencies for transformational leadership are intended to constitute a transversal qualification, emphasizing specific behaviors, rather than being taught as distinct units in a course.

To support the transfer of this framework to the real-world environment, we recommend educational curricula incorporate transformational leadership competencies as follows:

i. Transition from intended to emergent learning by complementing research and teaching with a practical transformation-based orientation [4].
ii. Support students along the trajectory of individual development by helping them to reflect and collaborate.
iii. Provide methods and tools to aid students in thinking systematically about transformative work in their particular contexts [1, 2, 4, 19, 25, 41, 45–47].

Our framework for transformational leadership can aid policymakers who want to 1) individualize education and training institutions or 2) provide necessary resources for innovative learning methods. The framework can also 3) be used as an instrument to guide efforts to strengthen transnational health systems by providing demand-driven education.

Public Health managers can use our framework to 1) determine transformational leadership competencies of the workforce, 2) to identify training needs, 3) mix and match teams by competencies, 4) develop job descriptions or interview questions, 5) design performance reviews and 6) continuous quality management. In practice, this is achieved by using the supplementary competency assessment tool (Supplementary File—Self-assessment Tool: Competencies for Transformational Leadership). This self-assessment can take place in a direct exchange in the form of an interview or anonymously via a survey. For this, the frequency of demonstrating competency-related behaviors (descriptors) is used as an approach to quantify the level of competency attainment by an individual.

### Unanswered Questions and Future Research

We still need to know more about how to design educational systems that cultivate qualified transformational leaders competent to enter the workforce. To further improve the Competency Framework for Transformational Leadership, researchers should test the self-assessment tool in real-world settings to ensure its suitability for determining competencies. Researchers should seek to define value ranges for the individual stages of each competency and refine its descriptors, they should also determine the importance and relevance of single competencies in various contexts. Then the next group of researchers can determine which curricular elements (e.g., experiential learning, access to networks and partnerships, mentorship, replacement programs) most successfully support students in obtaining the necessary tools, competencies, and know-how to meet challenges in their work as transformational leaders [6, 12].

### Conclusion

Using a Delphi process, we successfully developed a framework of 10 competencies and their descriptors for four categories and four stages of competency in transformational leadership for public health. Public health educators can use our context-specific and context-sensitive framework to determine the degree to which transformational leadership competencies are achieved and mastered, optimize teaching curricula, strengthen links between educational and workforce sectors, tailor curricula to specific contexts, and potentially stem the tide of emigrating graduates. The competency framework could be applied to leadership development in domains extending beyond public health. Professionals can use it to benchmark workforce performance and systematically reveal competency gaps the educational sector can then address.
