# Supplementary material for: Competencies for Transformational Leadership in Public Health—An International Delphi Consensus Study
Source: Int J Public Health. 2024 Feb 23;69:1606267. doi: 10.3389/ijph.2024.1606267 (PMC10935731; doi:10.3389/ijph.2024.1606267)
Supplement: Supplementary file 1 [file DataSheet1.pdf]

SUPPLEMENTARY MATERIAL

Competencies for Transformational Leadership in Public Health - An International Delphi Consensus Study

CORRESPONDING AUTHOR:
Barbara Maria Bürkin, MSc, Department Education & Training, Swiss Tropical and Public Health Institute, Kreuzstrasse 2, 4123 Allschwil, Switzerland, Tel: +41 61 284 8669,
Email: barbara.buerkin@swisstph.ch

Contents

1. Literature review results 2
2. Development process Competency Framework for Transformational Leadership 6
3. Expert panel participants 8
4. Analysis of expert feedback 10
5. Questionnaires 28
6. Terminology 30
7. Defining descriptors for competencies 30
8. Self-assessment tool: Competencies for Transformational Leadership 33
9. Personal development plan 38
10. Additional information 41

1. Literature review results

Table S1 Compilation of relevant competencies and allocation to initial categories Knowing, Doing and Being as well as Clusters

Field of Development		Competency	Reference
		**Doing**	
Doing	1	Empathy<br>Sensing others' feelings and perspectives, and taking an active interest in their concerns.	(1)
Doing	2	Organizational awareness<br>Reading a group's emotional currents and power relationships.	(1)
Knowing/Doing	3	Cultural and ethical awareness (new)<br>Understands and applies cultural awareness and sensitivity in communication with diverse populations	(2)
Doing	4	Cultural and ethical awareness (new)<br>Collaboration with diverse groups	(3)
Doing	5	Cultural and ethical awareness<br>Communicates strategically by defining the target audience, listening and developing audience-appropriate messaging	(2)
Doing	6	Cultural and ethical awareness<br>Catalyses change (behavioural and/or cultural) in the organization, communities and/or individuals	(2)
Doing	7	Cultural and ethical awareness<br>Acts according to ethical standards and norms with integrity and promotes professional accountability, social responsibility and the public good	(2)
Doing	8	Coach and mentor<br>Sensing others' development needs and bolstering their abilities	(1)
Doing	9	Coach and mentor<br>Clearly identifies and supports the roles and responsibilities of all team members, including external stakeholders	(2)
Doing	10	Coach and mentor<br>Facilitates the development of others as leaders	(2)
Doing	11	Inspirational leadership<br>Inspiring and guiding individuals and groups.	(1)
Doing	12	Inspirational leadership<br>Effectively plans the allocation of work tasks to achieve the goals set by the organization	(2)
Doing	13	Inspirational leadership<br>Develops job descriptions to assure staffing at different organizational levels, conducts hiring interviews and evaluates candidate	(2)
Doing	14	Inspirational leadership<br>Prepares and delivers outputs to facilitate communication within and between organizations such as meeting agendas, presentations, reports and project dissemination	(2)
Doing/Knowing	15	Inspirational leadership<br>Demonstrates knowledge of basic business practices, such as terms of reference, business plans, contracting and project management	(2)
Doing	16	Inspirational leadership<br>Develop capacity-building strategies at the individual, organizational, and community level	(3)
Doing	17	Builds organizational capacity for ongoing change and self-renewal	(4)
Doing	18	Inspirational leadership<br>Ensures the availability of professional development opportunities	(2)
Doing	19	Inspirational leadership<br>Inspires and motivates others to work towards a shared vision, programme and/or organizational goals	(2)
Doing	20	Inspirational leadership<br>Effectively manages people, most specifically by providing clarity on task responsibility, ensuring sufficient resources and training and providing regular feedback on performance	(2)
Doing	21	Inspirational leadership<br>Act as a role model, builds trust and demonstrates positive and engaging behaviour	(2)
Doing	22	Inspirational leadership<br>Creates change strategy that integrates people, process, and content needs, including how to change mindset and culture to support new business directions	(4)
Doing	23	Influence<br>Wielding effective tactics for persuasion.	(1)

Doing	24	Influence<br>Influence others to achieve high standards of performance and accountability	(3)
Doing	25	Influence<br>Works across sectors in local, national and international organizational structures	(2)
Doing	26	Influence<br>Builds, maintains and effectively uses strategic alliances, coalitions, professional networks and partnerships to plan, generate evidence and implement programmes and services that share common goals and priorities to improve the health and well-being of populations	(2)
Doing	27	Influence<br>Establish a network of relationships, including internal and external collaborators	(3)
Doing	28	Influence<br>Communicate an organization's mission, shared vision, and values to stakeholders	(3)
Doing	29	Influence<br>Communicates and shares information and responsibility effectively at different organizational levels to gain political commitment, policy support and social acceptance for a health goal or programme	(2)
Doing	30	Influence<br>Communicates facts and evidence effectively within the context of translating science and evidence into practice and policy for various actors in the system and populations of concern to increase the effectiveness of responses to risks, threats and damages to health	(2)
Doing	31	Influence<br>Communicates health messages (including risks to health) effectively (both in writing and verbally) through a range of modern media and social marketing to lay, professional, academic and political audiences	(2)
Doing	32	Catalyzes people's commitment and highest contribution to the change	(4)
Doing/Knowing	33	Conflict management<br>Negotiating and resolving disagreements	(1)
Doing	34	Teamwork<br>Working with others toward shared goals. Creating group synergy in pursuing collective goals.	(1)
Doing	35	Teamwork<br>Communicates with respect when representing professional opinions and encourages other team members, including community members and patients, to express their opinions and contribute to decision-making	(2)
Doing	36	Teamwork<br>Create a shared vision	(3)
Doing	37	Teamwork<br>Demonstrates practicality, flexibility and adaptability in the process of working with others, emphasizing achieving goals as opposed to rigidly adhering to traditional and commonly used work methods	(2)
Doing	38	Teamwork<br>Effectively leads interdisciplinary teams to work in a coordinated manner in various areas of public health practice	(2)
Doing	39	Teamwork<br>Develop teams for implementing health initiatives	(3)
Doing	40	Convinces others in an honest, respectful and sensitive manner in order to get them to go along with one's objectives. It can also be the desire to have a specific impact or effect on others (Influencing)	
Doing	41	Fosters an environment that will encourage professional and personal growth and the transfer of knowledge to future talent (Developing talent)	(5)
Doing	42	Works to build and maintain friendly, trustworthy and open internal and external relationships and networks with people who are, or might become, important actors in achieving strategic-related goals (Strategic Networking)	(5)
**BEING**			
Being	43	Emotional self-awareness<br>Recognizing one's emotions and their effects	(1)
Being	44	Emotional self-awareness<br>Demonstrates emotional intelligence with awareness of how one's own beliefs, values and behaviours affect one's own decision-making and the reactions of others	(2)
Being/Doing	45	Emotional self-awareness<br>Critically reviews and evaluates own practices in relation to public health principles, including critical self-reflection	(2)
Being	46	Emotional self-control<br>Keeping disruptive emotions and impulses in check	(1)
Being	47	Emotional self-control<br>Demonstrate a commitment to personal and professional values	(3)
Being	48	Adaptability<br>Flexibility in handling change	(1)

Being	49	Adaptability<br>Is able to cope with uncertainty and to manage work-related stress	(2)
Being	50	Adaptability<br>Actively prepares and adapts to changing professional environments and circumstances	(2)
Being/Doing	51	Adaptability<br>Delivers tasks within a limited time frame to be able to work with deadlines	(2)
Being	52	Achievement orientation<br>Striving to improve or meeting a standard of excellence.	(1)
Being	53	Achievement orientation<br>Demonstrates willingness to pursue lifelong learning in public health	(2)
Being	54	Achievement orientation<br>Self-assesses and addresses own development needs based on career goals and required competencies	(2)
Being	55	Achievement orientation<br>Acts on and promotes evidence-based professional practice	(2)
Being	56	Achievement orientation<br>Demonstrates an ability to understand and manage conflict-of-interest situations, as defined by organizational regulations, policies and procedures	(2)
Being	57	Achievement orientation<br>Shows entrepreneurial orientation through proactiveness, innovativeness, risk-taking, generating potential solutions to critical situations and evaluating their feasibility	(2)
Being	58	Positive outlook<br>Seeing the positive aspects of things and the future	(1)
Being	59	Positive Outlook<br>Demonstrates persistence, perseverance, resilience and the ability to call upon personal resources and energy at times of threat or challenge	(2)
	60	Sensitivity to social inequalities - empathy and ability to listen sensibly, with ethics and respect for human diversity	Other
**KNOWING**			
Knowing	63	Evaluate organizational performance in relation to strategic and defined goals	(3)
Knowing	64	Guide organizational decision-making and planning based on internal and external environmental research	(3)
Knowing/Doing	65	Align policies and procedures with regulatory and statutory requirements	(3)
Doing	66	Organize the work environment with defined lines of responsibility, authority, communication, and governance	(3)
Knowing	67	Perceiving multiple causal relationships in understanding phenomena or events	(1)
Knowing	68	Ensures that the change is aligned and integrated with all interdependent systems and processes	(4)
Doing	69	Implement strategic planning processes	(3)
Knowing	70	Uses conscious process thinking to design the change as a full stream process	(4)
Doing	71	Models and promotes the emerging mind set and way of being to the organization	(4)
Knowing	72	Perceiving themes or patterns in seemingly random items, events, or	(1)
Knowing	73	Understands the interdependence, integration and competition among health-care sectors and various actors who have interests in public health issues	(2)
Knowing	74	Understands the principles of systems thinking and can apply them within systematic enquiry to analyse, model and improve public health organizations and services at different strategic levels	(2)
Knowing	75	Identifies, connects and manages relationships with stakeholders in interdisciplinary and intersectoral projects to improve public health services and achieve public health goals	(2)
Knowing	76	Evaluates partnerships and addresses barriers to successful collaboration to improve public health services	(2)
Knowing	77	Creates and sustains conditions for success for the change, especially the continuous generation of new information	(4)
Knowing/Doing	78	Understands and applies effective techniques for working with boards and governance structures, including regulatory, professional and accreditation agencies	(2)
Knowing/Doing	79	Effectively applies knowledge of organizational systems, theories and behaviour to set priorities for, align and deploy all relevant resources towards clear strategic goals and objectives	(2)
Doing	80	Is proactive in designing and monitoring quality standards and applies quality improvement methods and tools to identify internal and external facilitators and barriers that may affect the delivery of the 10 EPHOs	(2)
Doing/Knowing	81	Effectively uses key accounting principles and financial management tools, such as financial plans and measures of performance	(2)
Doing/Knowing	82	Effectively uses risk management principles and programmes, such as risk assessment and analysis	(2)
Doing/Knowing	83	Understands and applies the principles of economic thinking in public health	(2)
Doing/Knowing	84	Performs health economic evaluation and assessment of a given procedure, intervention, strategy or policy	(2)
Doing/Knowing	85	Use informatics principles in the design and implementation of information systems	(3)
Doing/Knowing	86	Apply principles of human resource management	(3)
Doing/Knowing	87	Deploy quality improvement methods	(3)
Doing	88	Prepare professional plans incorporating lifelong learning, mentoring, and continued career progression strategies	(3)

Doing/Knowing	89	Applies methods, (digital) technologies and good practices for managing, analysing and storing data and health information	(2)
Doing/Knowing	90	Understands and applies a range of relevant information technology tools, social media and software	(2)
Knowing	91	Is able to identify patterns across situations that are not obviously related, and to identify key or underlying issues in complex situations (Analytical Thinking)	(5)
Knowing	92	Is able to generate results by assuming responsibility for one's performance and the correctness of one's interventions, and recognising opportunities and acting efficiently at the appropriate moment and within the given deadlines (Achievement Focus)	(5)
Knowing	93	Is able to respectfully communicate ideas and information (often technical) in writing to ensure that information and messages are understood and have the desired impact (Drafting Skills)	(5)
	94	Is able to effectively adapt to a variety of situations, individuals or groups. It is based on the ability to understand and appreciate different and opposing perspectives on an issue, to adapt an approach as the requirements of a situation change, and to change or easily accept changes in one's own organisational or job requirements (Flexible Thinking)	(5)
Knowing	95	Understands human, financial, and operational resource issues to make decisions aimed at building and planning efficient project workflows, and at improving overall organisational performance (Managing Resources)	(5)
Knowing/Doing	96	Working together effectively with interdependent goals and common values and norms to foster a collaborative environment and drive teams in the same direction (Teamwork and Team Leadership)	(5)
Knowing	97	Is able to understand internal/external clients' (e.g. Committees, working groups, country representatives, etc.,) needs and concerns in the short to long-term and to provide sound recommendations and/or solutions (Client Focus)	(5)
Knowing	98	Is able to hear accurately and understand unspoken, partly expressed thoughts, feelings and concerns of others (including cross-cultural sensitivity)	(5)
	99	Is able to keep one's emotions under control and restrain negative actions when faced with opposition or hostility from others or when working under stress (Diplomatic Sensitivity)	
Knowing	100	Works towards win-win outcomes. At lower levels, this competency assumes an understanding of one's counterparts and how to respond to them during negotiations. At the higher levels, the competency reflects a focus to achieve value-added results. (Negotiating)	(5)
Knowing	101	Understands the power relationships within the Organisation and with other organisations (including formal rules and structures, decision-making processes and influencers) (Organisational Knowledge)	(5)

Table S2 References used for compiling competency list version 1

(1) Competencies as a behavioral approach to emotional intelligence[1]	(2) WHO-ASPHER Competency Framework for the Public Health Workforce in the European Region[2]	(3) Core competencies for doctoral education in public health[3]	(4)Beyond change management: Advanced strategies for today's transformational leaders[4]	(5) Competency Framework[5]
1,2,8,11,23,33,34,43,46,48,52,58,60,67,72	3,5,6,7,9,10,12,13,14,15,18,19,20,21,25,26,29,30,31,35,37,38,44,45,49,50,51,53,53,55,56,57,59,73,74,75,76,78,79,80,81,82,83,84,89,90	4,16,24,27,28,36,39,40,47,63,64,65,66,69,85,86,87,88	17,22,32,68,70,71,77	41,42,91,92,93,94,95,96,97,98,99,100, 101

[1]Boyatzis, R. and R. E. Boyatzis (2009). "Competencies as a behavioral approach to emotional intelligence." Journal of Management Development.
[2]WHO (2020). WHO-ASPHER Competency Framework for the Public Health Workforce in the European Region, WHO Regional Office for Europe.
[3]Calhoun, J. G., et al. (2012). "Core competencies for doctoral education in public health." Am J Public Health **102**(1): 22-29.
[4]Anderson, D. and L. A. Anderson (2002). Beyond change management: Advanced strategies for today's transformational leaders, John Wiley & Sons.
[5]OECD (2014). Competency Framework.

2. Development process Competency Framework for Transformational Leadership

Figure S1 Initial version of the Competency Framework for Transformational Leadership (left) compared to the final version (right) following the consensus workshop

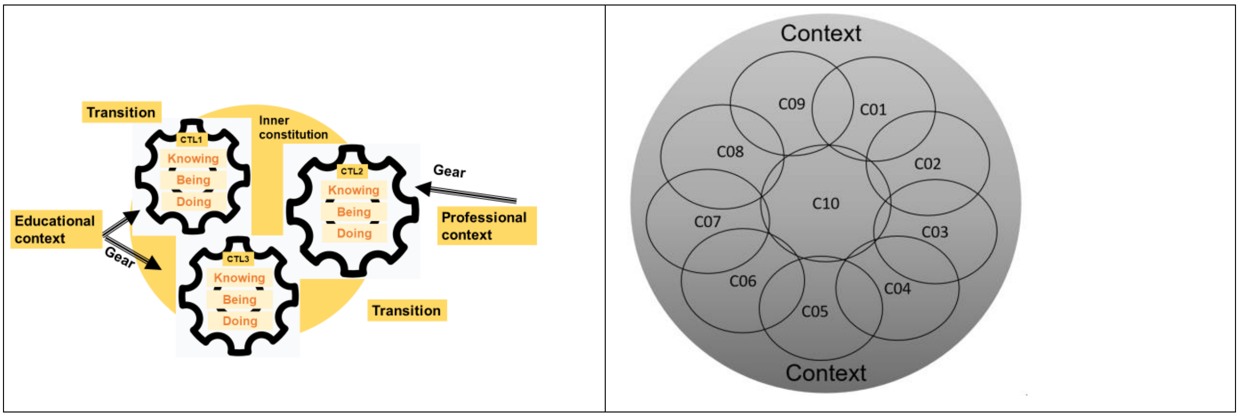

Figure S2 Advanced version of the Competency Framework for Transformational Leadership

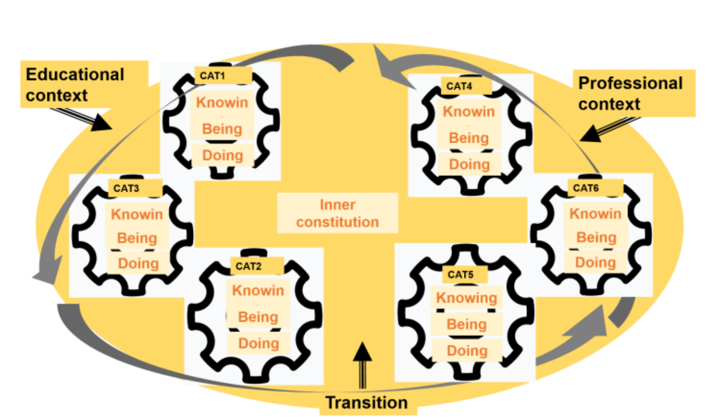

Category 1 (CAT1) = Process
Category 2 (CAT2) = Context
Category 3 (CAT3) = Self
Category 4 (CAT4) = Systems Thinking
Category 5 (CAT5) = Relationships
Category 6 (CAT6) = Mind-set

Box S1 Competency list version 1

1. Is able to identify patterns and underlying issues across situations and in seemingly random items. (91,72)
2. Is able to discern interdependences and power relationships within and outside the organisation (including formal rules and structures, decision-making processes and influencers) and addresses barriers to successful collaboration to improve public health services. (73, 76, 67, 101)
3. Applies principles of systems thinking within systematic enquiry to manage relationships with stakeholders in interdisciplinary and intersectoral projects and programmes. (74,75,79)
4. Guides organisational decision-making and planning in relation to strategic goals, based on internal performance evaluation and external environmental research (63, 64,66)
5. Uses conscious process thinking to design and implement strategic planning processes aligned with regulatory and statutory requirements and integrated with all interdependent systems. (70, 65, 68, 69, 65)
6. Applies principles of human, financial, project and operational resource management including risk assessment and quality improvement methods to efficiently organise project workflows and to improve organisational performance (15,80,81,82,83,84, 86,87,88,95)
7. Deploys (digital) technologies, good practices and social media to implement information systems and to manage, analyse and store data and health information (85, 89,90)
8. Continuously generates and communicates (new) information respectfully and effectively through a range of modern media channels to lay, professional, academic and political audiences.
9. Senses development needs and supports roles, abilities, and responsibilities of others, including external stakeholders (8,9)
10. Facilitates the development of others as leaders and teams for implementing health initiatives (10,39)
11. Fosters an environment including professional development opportunities that encourages professional and personal growth and the transfer of knowledge to future talents (18,41)
12. Develops capacity, including strategies at the individual, organisational & community level for ongoing change and self-renewal (16,17)
13. Effectively manages people, specifically by providing clarity on task responsibility, ensuring sufficient resources & training and provides regular feedback on performance (20)
14. Inspires, motivates, builds trust and guides others to engage and to work towards a shared vision, programme and/or organizational goal (11, 21, 36,19)
15. Creates change strategies (behavioural and/or cultural) and catalyses the emerging mind-set that integrates people, communities, processes, and content needs, to support new business directions (6,22,32,71)
16. Creates group synergy in pursuing collective, interdependent goals, common values and norms to foster a collaborative environment including respectful communication and participative decision-making (96,34,35)
17. Demonstrates practicality, flexibility and adaptability in the process of working with others, emphasizing achieving goals as opposed to rigidly adhering to traditional and commonly used work methods (37)
18. Effectively leads interdisciplinary and diverse teams by negotiating and resolving disagreements to work in a coordinated manner in various areas of public health practice (33, 38,4)
19. Effectively plans the allocation of work tasks including the development of job descriptions, interviewing and selecting candidates to achieve the goals set by the organisation (12,13)
20. Facilitates communication within and between organisations by delivering outputs such as meeting agendas, presentations, reports and project dissemination (14)
21. Communicates the organisation's mission and values to stakeholders and effectively shares information and responsibility at different organisational levels to gain political commitment and social acceptance (28, 29)
22. Communicates health messages, facts and evidence effectively and strategically by defining the target audience, listening and developing audience-appropriate messaging and within the context of translating science and evidence into practice (30,5)
23. Motivates others in an honest, respectful and sensitive manner to achieve high standards of performance and accountability (23,24,40)
24. Effectively works in professional networks and partnerships across sectors to generate evidence and to implement programmes and services based on common goals and priorities. (25, 26,27)
25. Applies effective techniques for generating win-win outcomes with people who might be important for achieving strategic-related goals (42,100, 78)
26. Recognises one's emotions and is aware of how one's own beliefs, values and behaviours affect one's own decision-making and the reactions of others (43, 44)
27. Critically reviews and evaluates own practices in relation to public health principles, including critical self-reflection (45)
28. Demonstrates persistence, optimism, perseverance, resilience and the ability to call upon personal resources and energy when delivering tasks within a limited timeframe or at times of challenge (51,58,59)
29. Self-regulates disturbing emotions and impulses and restrains negative actions when faced with uncertainty, work-related stress or opposition and hostility from others (46,49, 99)
30. Appreciates diverse perspectives on an issue and flexibly adapts to a variety of situations, individuals or groups (48,50,94)
31. Strives to meet a standard of excellence through proactiveness, innovativeness, risk-taking and by acting on evidence-based professional practice (52,55, 57)
32. Is willing to pursue lifelong learning including self-assessing and addressing own development needs based on career goals and required competencies (47,54)
33. Assumes responsibility for one's interventions by recognising opportunities and acting efficiently at the appropriate moment and within the given deadlines (92)
34. Acts according to ethical standards and norms with integrity including professional accountability, social responsibility and the public good (7)
35. Manages conflict-of-interest situations, as defined by organisational regulations, policies and procedures (56)
36. Senses power relationships and social inequalities and takes an active interest in others' feelings, perspectives or emotional currents (1,2, 60)
37. Demonstrates cultural awareness and sensitivity in communication with diverse populations including the understanding of unspoken, partly expressed thoughts, feelings and concerns. (3,98)
38. Appraises the needs and concerns of internal/external stakeholders (e.g. committees, working groups, country representatives, etc.,) in order to derive sound recommendations and/or solutions from this (97)

3. Expert panel participants

Table S3: Key attributes of Expert Panel participants across all Delphi rounds

		Round 1	Round 2	Round 3	Round 4	Round 5
**General**	Invited					
	Participated	**N=44**	**N=38**	**N=40**	**N=11**	**N=25**
	Finished					
	Response Rate	73%	63%	66%	6%	41%
**Gender**	Female	59.1%	60.5%		70%	
	Male	40.9%	39.5%		30%	
**Perspective***	Education	54%	54.1%	n/a	60%	n/a
	Labour market	0%		n/a	-	n/a
	Human resource or organisational perspective	11.4%	2.7%	n/a	-	n/a
	Research and Development	52.3%	35.1%	n/a	-	n/a
	Policy	20.5%	5.4%	n/a	20%	n/a
	Other	20.5	2.7%	n/a	20%	n/a
**Age**	Younger than 25	-	-	n/a	-	n/a
	25-35	2.3%	2.6%	n/a	-	n/a
	36-45	27.3%	23.7%	n/a	40%	n/a
	46-65	63.6%	68.4%	n/a	60%	n/a
	Above 65	6.8%	5.3%	n/a	-	n/a
	Prefer not to say	-	-	n/a	-	n/a
						**Nationality**
**Africa**	Ghana	1	1	n/a		n/a
	Kenya	3	2	n/a		n/a
	South Africa	4	4	n/a		n/a
	Nigeria	2	2	n/a		n/a
	Togo	1	1	n/a		n/a
	Uganda	2	2	n/a	1	n/a
	Malawi	-	1	n/a		n/a
**Americas**	USA	2	1	n/a	1	n/a
	Argentina	1		n/a		n/a
	Brazil	1	1	n/a	1	n/a
	Columbia	2	1	n/a		n/a
	Canada	1	1	n/a		n/a
	Mexico	3	2	n/a		n/a
	Costa Rica	1	-	n/a	1	n/a
	Chile		-	n/a	1?	n/a
**Europe**	UK	3	1	n/a		n/a
	Denmark	1	1	n/a		n/a
	France	1	2	n/a		n/a
	Germany	1	1	n/a	1?	n/a

	The Netherlands	1	-	n/a		n/a
	Spain	2	2	n/a	2?	n/a
	Switzerland	4	5	n/a		n/a
	Ukraine	1	1	n/a		n/a
**Asia**	China	1	1	n/a		n/a
	India	1	1	n/a	1?	n/a
	Laos	1	-	n/a		n/a
	Pakistan	1	1	n/a	1?	n/a
	Thailand	1	-	n/a		n/a
	Vietnam	-	1	n/a		n/a
	Mongolia	1	-	n/a		n/a
**Total: 30 Countries**						
						**Workplace**
**Africa**	Ghana	1	1	1		1
	Kenya	4	3	3	1	2
	South Africa	4	5	3		
	Nigeria	2	2	2		1
	Togo	-	-			
	Uganda	2	2	2	1	1
	Malawi	-	-			
	Cote d'Ivoire	1	1			
	Tanzania	1	1	1		1
**Americas**	USA	4	2	3	1	3
	Argentina	1	-			
	Brazil	1	1	1	1	1
	Columbia	1	1	1		
	Canada	1	1	1		1
	Mexico	1	1	1		1
	Costa Rica	1	-	1	1	1
	Chile	-	-	1	1	
**Europe**	UK	1	-	1		1
	Denmark	2	1	1		
	France		-			
	Germany		-			
	The Netherlands		-	1		1
	Spain	1	1	1	1	1
	Sweden		1			
	Switzerland	8	8	8	1	7
	Ukraine	1	1	1		
**Asia**	China	1	1	1		
	India	1	1	1	1	1
	Laos	1	-	1		
	Pakistan	1	1	1	1	1
	Thailand	1	-			
	Vietnam	1	1			
	Mongolia		3	1		

*Multiple answers possible

Box S4 Further perspectives and experiences mentioned by experts

Round 1	Round 2
• Consultancy<br>• Foundation/Philanthropy<br>• Funder of research capacity strengthening within the African higher education environment<br>• I am working mostly at the project focused on medical education (that is project management + policy + org perspective) as well as I am educator at the university and I am conducting, supervising research within or outside the project and the university<br>• Medical and International Health Fields with emphasis in communicable diseases<br>• Public Health<br>• Research Capacity Strengthening<br>• Voluntary work - currently chair two Boards of Directors; also do volunteer teaching and project work in Costa Rica, where I spent 6 months a year.<br>• Control programme	• Health

4. Analysis of expert feedback

Box S6 Newsletter Round 1: Delphi Study on competencies for transformational leadership

Newsletter Delphi Round 1

Dear Experts,

A few weeks have passed since the first Delphi round. Since then, we have been working intensively on the data analysis. We have been overwhelmed by the richness and wealth of feedback we have received. ***Thank you so much! :-)*** *Before the second questionnaire round starts from* ***01-15 September 2021****, we would like to share with you a first newsletter in which we have summarised topics that were mentioned repeatedly as* ***'Golden Nuggets'*** *and provided examples. We ask for your understanding that we could not include all answers, although we considered them to be of great relevance. The selection of the examples and presentation of the content was made on the basis of the frequency in which a particular topic was mentioned.*

In case you are interested please find below the steps we have taken to analyse the data:

Step 1
Listing of all competencies and ranking according to the degree of agreement (threshold 85%).

Step 2

Analysis and selection of relevant text passages within open-ended responses (2.1 each competency 2.2 each category (Being, Knowing and Doing) and 2.3 comments and recommendations for the list of competencies (end of survey)).

Step 3
Implementation of a qualitative content analysis including the development of a coding frame (categories, definition, indicators and examples).
Identification of the following superordinate categories for transformational leadership: 1) Process 2) Context 3) Self 4) Systems thinking 5) Relationships & 6) Mind-set.

Step 4
Consolidation of the quantitative and qualitative results.

Step 5
Carrying out statistical calculations.

In addition a calendar invitation to participate in the ***Online Consensus Workshop*** *on* ***19 October 2021*** *will be sent out shortly after this information.*
Stay tuned for the second round!

Barbara
On behalf of the research team

****

Golden Nuggets

Nugget 1: Non-linear processes and the application of tools are of paramount importance for transformational leadership.
Example comment on C01**:** *«In the African context, there is a tendency to assume a linearity to transformation processes. Transformational leaders, whether as researchers or leaders of research organizations, can see beyond the superficial and obvious, and get to the underlying issues to solve deep, often structural, problems»*

Example on C05: *«Sometimes the requirements expressed are hurdles that must be cleared rather than enablers of progress. Transformational leaders will be able to walk the line between flaunting regulations and getting bogged down in them.«*

Comment on C07: *Seeks out and applies innovative social and digital technologies to lead systems innovation and social transformation*

Nugget 2: «Transformational» is about the way one acts on knowledge in a specific context.
Example comment on category KNOWING: *«Knowledge on the objectives of society (target knowledge) should be more prominently mentioned. (...) it is also about understanding the actual (local) development context, its problems, the stakeholder's power and interests, and not least local approaches or solutions to bring about (positive) change.»*

Example TL experience of one of the participants (C09): »*In my organisation at my university my head of school has changed the chairing of our school admin meetings that occur once a month. Now instead of the Head of school chairing it rotates, what makes it transformationalis that it includes ALL staff, also admin staff, not only academics. What evidence have I of it being transformational? Everyone has been impressed that all staff can do this. Also last month the person doing it was a post grad coordinator (note in here these people may not have a tertiary degree) and he asked the Head of School why he chose to run the meetings that way. This was a demonstration of a change in usual hierarchical relationships. A junior less educated member of staff asked the most senior person about his motivation for choices he made in leading the school in a public forum.»*

Example comment on category KNOWING: «[*TL] is about practice and learning by doing which depends (…) on the context (country, field)... (that is 'doing') ...»*

Example comment on C07: «*Depends on the environment in which you work, you can have this competency but if no technologies [are available] (…) you can not use it.»*

Example comment on C07: *«(…) Understanding the limitations of digital technology is as important as understanding the power of it.»*

Example comment on category KNOWING: «*Knowing (…) is more context based (…) for example, for health financing - it is knowledge of health financing, for medical education - it is understanding how medical education as system works etc.»*

Example comment on C31: «*EXCELLENCE doesn't exist. One has limitations or limits and the projects that one works has limitations too (time, financial resources, workforce limitations) (…).»*

Example comment on category KNOWING: «[*TL] is about practice and learning by doing which depends (…) on the context (country, field)... (that is 'doing') ...»*

Nugget 3: Transformational leadership is not about becoming a «martyr» but about «Self-care».

Example 3 comment on category DOING: «(…) *One is not perfect and perfection does not exist. One can make mistakes at the personal and/or professional life, and these mistakes may affect one's reputation. An important competency is to prevent mistakes and mitigate the impact of them.»*

Example comment on C39: «(…) *Not all transformational leaders are able to self-regulate on their own, and may need counseling in difficult circumstances. For example, a leader of an organization constantly facing criticism for not being sufficiently racially sensitive. Though the person tries her best to be so, she may require additional skills which can only be provided by an outside professional* (…) Operating from vulnerability»

Example comment on KNOWING: «(…) *Knowing what we dont know and therefore need to call on others to be our allies, partners, collaborators, our sources of evidence, our citizens on the barricades and the people we defer to and follow when they know more , and have more traction on the issues we are concerned about»*

Example 2 comment on C28: «(…) *The conundrum and the challenge of leadership – (…) to demonstrate all these qualities- even when you are exhausted, drained, exposed, weak, tired, and you have no personal resources left-there is no-one else to complain to ! you and those resources -that's the job!»*

Example 1 comment on C28: «(…) *also depends on the outside conditions under which you work. One needs to be mindful about stress (…) it is ok to say no and change the situation. That is (…) transformational leadership*». No need to always be optimistic and cope.

Nugget 4: Transformation requires embracing diverse realities and their interplay in different contexts.
Example comment on C03:*«Systems thinking is essential to problem solving in fast-changing, complex environments.»*

Example comment on category KNOWING: *«(…) systemic knowledge needs to be added, by explicitly assuming an interaction perspective for addressing multiple development goals, which are related to the concerned health issues (see the whole discussion around the SDGs) (…) [A TL] has the big picture, integrates and addresses the determinants of the issues»*

Example comment on category KNOWING: **«***Knowing how to recognise challenges in the system (…) in order to move across actors in the system to face them. A principle challenge for young transformational leadership is that the 'heads of the units' / ' big bosses' are the top of the system (…) one needs to recognise challenges in the organisation/system and one needs to be smart to move in the system.»*

Example comment on category KNOWING: **«***Knowing how change happens in complex systems (…) knowledge on systems innovation practices - how to unlock systems (…) knowledge and ability on how to engage with social entrepreneurs and social innovators how to apply participatory leadership methodologies (see Art of Hosting Community) - to co-create and innovate».*

Example comment on C05: «*(…) Sometimes you may not need all the interdependent systems. You may need a few depending on the task being implemented.»*

Nugget 5: Communication and relationship building are the scaffolding. However, this is not about win-win situations, but about transformation itself.
Comment on category KNOWING: «(…) [TL] *understand the power relations that are at play (…) within own group (race, class, gender, nationality etc.) and applies this knowledge in how you lead. To have a political and economic analysis of the area your work is focused on and to use this in the way you direct your work.»*

Comment on category KNOWING: *»Understanding differences in cultural practices and gender needs of staff in the organization.»*

Example comment on C08: «*Respect differences in the audience, an academic is different to the lay public or (…) even politicians. It would be a mistake to confuse these different stakeholders as customised, communication strategies are needed.»*

Example comment on C22: *«(…) The effective leader might have others help them (…)*

Example on C20: *(…) the leader leads and the team does the work. Their is the risk of micromanagement and missing on delegation.»*

Example comment on 07: «*A transformational leader (…) understand the role of technology, communications, and data/information in producing excellent research and ensuring that decisionmakers at all levels have access to accurate information. The transformational leader might not be able to do this work themselves, but (…) ensures that someone on the team can.»*

Example comment on category KNOWING: *«[Promotes] professional growth and equity. Knowledge of tools to assess, measure and quantify employee inputs so as to adequately assess employee competence and fit for promotions. (…)A key roles of a TL is to create more outstanding professionals and one of the key driving factors in that role will be an effective and transparent reward system that actually rewards competence and creates healthy competition.»*
Example comment on category KNOWING: *«[TL]* use technical knowledge to intellectually stimulate others to perform».

Nugget 6: There is no recipe for transformational leadership, merely the ability to act from an authentic inner place.
Example on categroy KNOWING: (…) «[TL is about attitude], *transformational leaders care about the state of things and feel entitled and responsible to change it. Otherwise, they won't get to do anything»*

Example comment on C05: (…) *Not possible to limit process thinking to conscious. (…) a lot may be unconscious, likely being influenced by one's previous experiences, attitudes and* cultural concepts.

Example comment on Category KNOWING*: «Perhaps emotional intelligence which involves sensitivity to others' feelings and reactions, and should help to guide our work. For example, a young Muslim student sits behind her screen for her Zoom classes with a hijab, black background and surrounded by a gloomy atmosphere. She doesn't speak unless drawn out by the professors, and when she does, her answers are excellent. If the professors were not emotionally intelligent, they would give her a very low mark on class participation, which would be emotionally and culturally insensitive.»*

Example comment on category KNOWING: *«(…) The obvious is knowing one's field exceptionally well (subject competence). Also, knowing what you don't know, and knowing how to identify talent to fill your own competency gaps (e.g., technology, social media).»*

Example comment on C38*: A transformational leader will do this out of instinct» « to transform may be to obstruct/change power relations and then someone loses and it is not a win win at all»*

Nugget 7: A single competency consists of the components Doing, Knowing and Beeing and cannot be divided into different categories.
Example comment on C37: «One can have cultural awareness but must also act on that awareness and demonstrate it (…) Being alone is not enough [one] also need to act on in «DOING»)

Example comment on C01: «Knowing cannot be disconnected to Doing and Being is socially and culturally constructed»

Example comment on category KNOWING: «(…) most competencies are in the domain of DOING & BEING. BEING competencies are of higher hierarchy, they include already the lower KNOWING competencies.»

Box S7 Newsletter Round 2: Delphi Study on competencies for transformational leadership

Newsletter Delphi Round 2

Dear Expert,

The second Delphi round is completed and we have spent the last weeks analysing your valuable feedback to now come up with an adapted list of competencies.
Thank you for your participation and all the constructive suggestions and comments!!

*As we did after the first round, we have summarised frequently mentioned topics as **'Golden Nuggets'** and added corresponding examples. Please find them below. Again, we ask for your understanding that we could not include all answers, although we considered them to be of great relevance.*

*If you have not yet registered for the **Online Consensus Workshop on 19 October 2021 from 12:00pm-3:00pm (GMT+2)** you are welcome to do so by Friday 15 October.*
The programme and dial-in details (zoom link) will be shared with all participants in advance.
I am looking forward to an exciting discussion! :-)

Barbara
On behalf of the research team

***

Golden Nuggets

Nugget 1: The chosen verb form is of central importance, as it reflects the complexity and thus the transformational character of a competency.

Example comment on C05:
"Is it 'strive for' or 'identifies' (more of a natural or learned/trained ability rather than an constant active search for it)."

Example comment on C09:
"This competency tries to incorporate too many ideas. Further, "striving" implies some kind of activist or entrepreneurial approach. Slightly reformulated, this would be a very-to-extremely important competency."

Example comment on C09:
"It is not about challenging relationships but rather about engaging and addressing."

Example in the comment and recommendation section:
“Some of the competencies are more a code of conduct or guidelines - some of the competencies could be lumped together”

Example in the comment and recommendation section:
“Some of the wording has to be revised that they are at the advanced level demonstrate from example .... rather to judge ...advise ...

Nugget 2: Self-regulation is different from being emotional and operating from vulnerability.

Example comment on C05:
(…) My problem here is that there are two questions - beyond a shadow of a doubt I think that recognising emotions is important if and when I let them interfere with my work. But is this related to self-care? I don't know that the two are really related. You can be a transformational leader and overwork and practise no self care and you can be very successful and you never burn out. Others may burn out. Also I have worked with transformational leaders who have achieved a lot and they are very thick skinned and I think do not recognise their own emotions or anyone else's. They are very successful and it may be because they are emotionally blunted. I also suspect this is a gender issue. In my experience men spend less time feeling and worrying about how other people feel and woman do it too much.”

Example comment on C07:
“I am not convinced that a leader should operate out of vulnerability. It may be that I am not aware of what operating out of vulnerability actually means.”

Example comment on C07:
“Operates out of vulnerability could be open to misinterpretation - perhaps operates authentically acknowledging vulnerability”.

Example comment on C07:
“(…) self regulation is hugely important - BUT is it related to vulnerability? I don't think so. I have seen people who operate out of vulnerability who fail just because of that reason. I have also seen people who are seen to be very strong and invulnerable succeed, usually because they seem to be confident and sure of themselves and have no doubts or vulnerabilities. But I have also seen them succeed because they sometimes do very rarely demonstrate / know / expose a particular vulnerability. So I agree that self regulation is important but I would not link it to vulnerability.”

Example comment on C07:
(…) I would not restrict it to operating out of vulnerability; it is important to engage with ones own vulnerability and establish authentic communication, but for some people operating out of it conflicts with their own way to handle issues in a healthy manner, not everyone engages with vulnerability in the same manner

Example comment on C07:
“Checking the ego is important”

Nugget 3: One’s character is key for transformational leaders.

Example comment on C08:
"(…) this may fall a bit within the purview of individuals' character which might be an asset you will want to look for as it is otherwise difficult to groom. Excellent human resources competencies would be a must.

Example comment on C08:
"You may need to give this more thought. Leaders who don't have this competency cannot be transformation, if only because transformation is extremely difficult even in less challenging times."

Nugget 4: A transformational leader will never be able to please everyone / Transformation implies change and it isn't conflict free.

Example comment on C11:
"I do think this is very idealistic and hard to achieve - there is always someone in a group who will find the leader insensitive, or will feel disrespected even if that was not the leaders intent. But this is certainly something to aim for."

Example comment on C12:
"(…) Bear in mind that a decision has to be made, even if a full consensus isn't possible."

Example comment on C12:
"(…) just to point out if you are trying to force change there are always people/institutions with vested interests who will try to obstruct the transformation you seek to achieve. (…) you work actively to see that some view points are not taken into account and those groups/people will not think that you have built trust, even though you may have with the majority. The idea that transformation/ change is devoid of conflict is naive and sometimes I feel these statements suggest that this is a wonderful process where everyone is taken care of and accounted for and considered and that is not that case. If you want greater equality then those with power have to give up something, if you want to change an organisation so that it is more efficient the lazy people or usual systems have to be chucked out or changed. (…) participatory decision making is right but it can sometimes go with lack of trust at least for some.

Example comment on C13:
"Sometimes if you are looking to change something for the general good you can be in the minority - so you would never co-create with everyone - only particular stakeholders who have the same mission - sometimes some of the stakeholder you have to engage with are one you do not build a coalition with - you form a coalition with others against an obstructive stakeholder."

Example in the comment and recommendation section:
"What is missing is a recognition that transformation is about change, and change is not always comfortable and it is not conflict free (…).

Nugget 5: Transformational leadership cannot only focus on Public Health, it is about seeing the whole picture.

Example comment on C11:

"I think we should not restrict ourselves to public health, there are other way more important topics such as climate and biodiversity collapse"

Example in the comment and recommendation section:
"(…) You need to broaden the scope of scenarios beyond what we, honest individuals and leaders would like to see to scenarios which are much more complex and strained as a result of global warming, the pandemic and its brutal economic impact. Among the biggest challenges future public health leaders will face is how to change people's minds, perceptions, attitudes towards the impact of the global crisis, change corporate culture and address an ever increasing corruption phenomena."

Nugget 6: Integrity, trust and acting ethically are above the importance of transparency.

Example comment on C11:
"(…) in transformational strategies it is important that you may not be 100% transparent all the time. (…) however (…) integrity is paramount. And it is very important to be ethical and super important that people groups feel they are treated the same. So trust is important. Linking all of this with transparency is problematic."
Example in the comment and recommendation section:
"I would say that however that integrity is super important. I have certainly followed and changed my practise / process when I have not agreed with a leader but I have found there is evidence or rational argument to support that change and that the person has integrity. But not just any integrity, but that is based on a value system that I support."

Box S8 Explanation for reformulation/adaptation of competencies

Adaptations after Delphi Round 1 (EvaSys Survey)

C01:
- Important to specify random items (seven times mentioned)
- All categories, "trait" or "state" issue
- ***"Able to identify patterns and root causes across situations and systems OR in different contexts and situations"***
- ***"Able to understand the underlying causes of complex issues and apply pattern recognition to problem solving"***

C02:
- 3 comments suggest to split into 2-3 different competencies,
- Addresses both doing and knowing "***barriers and opportunities to successful engagement to..."***
- 1 = *"**Is able to discern interdependences and power relationships within and outside the organisation (including formal rules and structures, decision-making processes and influencers**)"* and
- 2 = *"**addresses barriers to successful collaboration to improve public health services"***

C03:
- Different category (Doing/ Related to mindset or behaviour)
- Transdisciplinary and interdisciplinary both relevant
- Topic of 'systems thinking', overarching competency? C03 contains C02&C01

- Mention context or just skill?
- ***"a catalyst of intersectoral multi-stakeholder projects and programs"***
- ***"Applies principles of systems thinking within systematic enquiry to influence relationships with stakeholders in interdisciplinary and intersectoral projects and programmes."***
- ***"Applies principles of systems thinking to manage relationships with stakeholders in interdisciplinary and intersectoral projects and programmes."***

C09:

- Category knowing and doing together (3 times), all categories
- 'Senses' not the right verb (3 times), instead appraises, identifies (3 times), perceives
- Internal and external stakeholders are different (2 times)
- What is the transformative essence of competency?
- To sense something is to know it - it is not about doing anything - to do you would have to put something into action with it and what makes this transformational is about how you act on that sense. And I think that it is context specific, so a good leader (transformational of otherwise) may have this sense but it is how they act on it that makes it a DOING thing and if it transformational or not will be context specific.
- "Creates opportunities for staff and partners to learn together by experimentation: co-creation, rapid prototyping and implementation, followed by adaptation (change is iterative)."
- "…senses and respond to"
- "Identifies development needs, including those that relate to roles, abilities, and responsibilities and strategizes on how to address them"
- "determine development needs of internal stakeholders and supports capacity building on these"

C10:

- Includes a teaching component, (mentioned by two persons), requires active listening and trust, mindset of a leader
- More than health initiatives, also include economic and social aspects
- 2 competencies in one "***Develops leaders"*** and 2***) "Develops teams".***
- Not clear hat is the "essence of transformational is"
- possibly combining 9 & 10

C11

- Not only knowledge transfer but also knowing, doing, being
- Competency between Doing and Being, requires "to be" Again teaching component
- "***Sets up innovation labs for learning, stimulates experimentation***"
- ***"Fosters professional and personal growth within the team/next generation***"

C12:

- Too complex, operationalisation
- Context-specific, not always on all levels, relevance to community work depends on the nature of the organization and the role of the leader (two times)
- Without "including strategies"
- Essence of transformation is not clear
- ***"Develops capacity at the individual, organisational & community level for purposeful/mission driven change and innovation"***
- ***"Develops capacity at the individual, organisational and community level through multiples strategies to achieve ongoing change and renewal."***

C13:

- Between management and leadership, this competency is not specifically for TL (four times mentioned)
- Too many aspects
- Being component
- Just one aspect; 'manages people enabling them to deliver'; or, 'Provides clear expectations, well

- defined responsibilities, and the means to deliver'
- ***"Applies participatory and agile practices to stimulate performance and impact of the organization, bringing a sense of wholeness to the way people work together"***
- ***"Effectively directs the organizational strategies, specifically by providing clarity on the strategies, related tasks and expected performance."***

C14:

- "Builds trust"… more important than the rest
- Shares Doing & Being
- Captures focus/managerial skills
- Two contradicting comments; one says this is a typical leadership quality, the other says this transformational leadership
- Needs to be manifested in leaders
- ***"Provides leadership to accomplish the corporate goals"***.

C16:

- Too complex, operationalisation (three times)
- Just good business or transformational?
- Building a high functioning team, Include: "***Managing diversity"***
- "***Foster collaborative environment through trust and participatory leadership methodologies"***

C17:

- too complex, operationalisation
- competency includes a value judgement, this is problematic
- no need to negate a behaviour when something is already confirmed
- process to achieve goals is also important
- Contradiction: one says this competency is not transformational, the other says this is clearly transformational
- ***"Demonstrates practicality, flexibility and adaptability in the process of working with others and continuously challenges established ways of working/status quo to stimulate creativity and innovation"*** (in fact I think these are two separate competencies)
- ***"Demonstrates practicality, flexibility and adaptability in the process of working with others, emphasising achieving goals"***
- ***"Has a practical approach to problem solving" "Is driven and adapts to changing circumstances"***
- ***"…achieving <u>shared</u> goals"***

C21:

- Too complex, operationalisation
- Without word "political", not only organisations but also teams, projects etc. to be more inclusive; stop after levels,
- Competencies are role specific in organisations
- what is the essence about transformation, this is more a strategy
- a leader needs to have a vision (…) and deliver it
- ***"Communicates the organisation's mission and values to stakeholders and effectively shares information and responsibility at different organisational levels"***
- ***"Communicates the organisation's mission and values to stakeholders and effectively shares information responsibility at different levels to gain political commitment and social acceptance"***

C23:

- Too complex to operationalise
- Between doing and being
- Function of who the leader is (honest, respectful and sensitive)
- Contradiction as one comment says this is crucial and brilliant for TL whereas two other comments say that essence of Transformational is missing
- "***Is value-driven inspiring leader that strives for excellence***"
- ***"Practices participatory leadership methodologies to empower others to fully capitalize on other's competencies and collective wisdom"***

C26:

- Difficult to operationalise
- Knowing and being
- practices awareness based mental agility - 'responds instead of reacts'
- ***"Recognises one's emotions and is aware of how one's own beliefs, values and behaviours affect one's own decision-making and actions, and the reactions of others"***
- ***"Demonstrates high level of emotional intelligence, self awareness and active listening, applying inspirational value-driven leadership"***

C27:

- Being not so clear-cut, also doing, based on knowing (two times mentioned) rather an ongoing process, helps leader to stay on track especially at higher positions
- Again '**awareness based mental agility'**(see C26)
- Define what public health principles are
- ***"Applies awareness-based practices (e.g. meditation) to review and evaluate own practices..., while paying attention to self-care"***
- ***"Critically reflects on own practices."***
- ***"Critically reviews and evaluates own practices in relation to public health principles"***

C28:

- Aspect of "self-care" (three times mentioned), also an opposing voice that says that this is exactly the challenge and "conundrum"
- Too complex, operationalisation
- Perseverance only if there is flexibility and adaptability

C29:

- Typical leadership skill, not really transformational
- Stop after ***"***actions"
- Not helpful to suppress feelings
- To reach out for (professional) help (mentioned two times) "***Operating from vulnerability"*** as a key transformational leadership competency
- Quantifying disruptive behaviours on TL is not scientifically measurable. Correlation cannot be ascertained.
- ***"Avails of mechanisms for self-regulation of....."***
- ***"Acts empathetically, is resilient and patient."***

C30:

- Split into 2, and stop at issue for first part
- Consider to combine with valuing diversity
- Aspect of women in key leadership positions
- Underestimated competency for management situations, central in teaching for TL, should be part of educating TL
- ***"Adapts to diverse perspectives in varied situations"***
- ***"Is empathetic and humble, gets the best out of each person, valuing the contributions from every team member"***
- ***"Purposefully invites diverse perspectives and opinions, Celebrates diversity and inclusiveness through participatory leadership practices"***

C34:

- 3 times mentioned that this competency is critical/weighs more than others "***…and challenges the status quo where social injustice prevails***"
- "***Acts ethically***…"
- "***Is a paragon of integrity, fairness, and social justice / Is an inspirational leader admired for his/her fairness, integrity, and transparency***"

C36:

- Two different competencies 'power relationships' and 'social inequalities'

- Aspect of social and other inequalities and inequities (two times)
- Contains being empathetic, a good listener and fair
- Need that people need to feel uncomfortable to change
- Includes both, Knowing and Being
- "***Actively senses power relationships that maintain the social inequalities and applies and activist and entrepreneurial spirt to address them"***
- ***"Recognizes power relationships and social and other inequalities and actively attends to others' feelings, perspectives and emotional triggers"***

C37:

- Too complex, operationalisation
- Stop after "diverse populations"
- A "Being" competency, also Doing because one must act on awareness, hence demonstrate it; Doing because it is communication and Knowing because of cultural awareness
- "Must be contextually conscious"
- **"Acts with respect and professionalism in multicultural environments"**
- **"Cultivates deep listening practices and participatory leadership methodologies to invite diversity and inclusiveness into the development of systems for health"**

Adaptations after Delphi Round 2 (EvaSys Survey)

C01:

- Adaptability and flexibility close in meaning
- Verb "demonstrate" was replaced since it is regarded as a higher level competency
- Flexibility and Adaptability not always the "Gold standard", rather important to push for change
- C04 was included in this competency since it was mentioned five times that C04 would be redundant and should rather be part of C01

C02:

- It was mentioned five times that it would be required to separate the competency into different components

C03:

- The part external stakeholders was removed from the formulation since it was five times mentioned that this too resource-intensive and impossible. The competency was merged with competency C11.

C06:

- The part about emotions was removed since it was mentioned that recognizing emotions would not necessarily be related to self-care
- Some TL would not practice self-care and would never burn out, it would rather be about being emotionally blunted.

C08:

- Verb "demonstrates" was replaced since this wasn`t regarded as an advanced competency
- The suggested reformulation was adopted since it has a clearer focus on challenging situations and change, which are core characteristics of transformations

C10:

- Building trust not necessarily in the interest of a common goal, therefore the word trust was removed

C11:

- "How" was mixed with competency itself, restriction to public health was removed.

C12:

- Participative decision-making was regarded important but many comments referred to the fact that it would not always be possible to maintain trust for everyone involved.

- “Through” was removed as it was mentioned that this would mix up the how with the competency itself.

C13:
- Not limited to health, often mentioned, that the whole (system, ecology, climate change, etc.) need to be linked to it, Co-creation with all is not possible, there is always someone who doesn’t like change.
- Too many aspects in one competency, TL don’t build coalitions, rather initiates, monitors and measures, no co-creation alone.

C14:
- The word paragon was removed as it was twice not well-understood by the participants.
- Transparency was removed as it was mentioned that this may not be possible and that it would be problematic to link it to the other concepts.
- It would rather be about having a clear strategy, acting with integrity and being ethical, what leads to trust.

Explanation for inclusion/exclusion (competencies below the threshold 80%):

- C04: part of C01, therefore C04 was removed in its original formulation. Adopting to all situations and being overall flexible was not regarded as a must.
- C09: regarded as too complicated, therefore it was split into two parts. Challenging interdependencies was rejected, rather should they be challenged
- C05: was regarded as an intuitive/unconscious competency, something that TL do out of instinct. The original formulation was removed. It was suggested to link it to the competency on systems thinking (C02)
- C15: Not understood well as a competency (four times). It was suggested to include into the competency on systems thinking (C02)
- C07: Removed in its original formulation since many commented that they would not understand the competency formulation. Self-regulation was regarded as important but not linked to vulnerability (four times mentioned). Suggested reformulations were merged with C06

Adaptations after Delphi Round 3 (Consensus WS)

C01:
- Remains at it is, no major comments.

C02:
- No major comments during workshop. → Suggestion of a research group member was adopted.

C03:
- No major comments during workshop. → Suggestion of a research group member was adopted.

C04:
- No major comments during workshop. → Suggestion of a research group member was adopted.

C05:
- No major comments during workshop. → Suggestion of a research group member was adopted.

C06:
- No major comments during workshop. → instead of collaborative the term “diverse” was set, since collaborative is part of C09 (Practices participatory leadership)

C07:
- No major comments during workshop. → Suggestion of a research group member to simply sentence was adopted.

C08:

- Comment of one panelist to consolidate two into one (C08/C12) → The two competencies were merged.
- One of the competencies is now seen as the descriptor of the other.

C09:
- No major comments from the group. → Suggestion of a research group member was adopted.

C10:
- No major comments from the group. → Suggestion of a research group member was adopted.

C11:
- Suggestions for rephrasing of one of the research group members
- Comment of one panellist that Nourishing D&I is enough in itself.
  → Suggestion of research group member and panellist were adopted.

C12:
- No major comments from the group.

C13:
- Comment of one panellist that it would be the most ambiguous of all, word context needs to be considered.
- Comment of one panellist that it would need to be defined within the public health context.
- Comment of one panellist that this relates to human relations.
- Comment of one panellist Leadership can challenge unequal distribution of power, but not power itself.
  → Comments of panellist were adopted.

C14:
- Comment of two panellists that it would be difficult because of the word entrepreneurial.
- Comment of one panellist that the relation to public health should be made in the operationalisation
  Suggestion of panellists was adopted.

Excerpts from expert comments relevant for the reformulation/adaptation of framework

Gender:
- *It has a gender bias as well, to know about the background, family situation of different persons* (Panellist)
- *Self also requires Mind-Set, maybe also a gender issue* (Panellist)
- *To challenge power relations is not the same thing whether you are a man or a women* (Panellist)

Systems thinking and context:
- *Context is important because of the environment (…) context and relationships have a synergistic environment* (Panellist)
- *Context is about the ecosystems in which action take place* (Panellist)
- *The best way to train a transformational leader is holistically from the different aspects of the system* (Panellist)
- *Context has to do with adaptations* (Panellist)
- *To do adaptations properly it needs systems thinking* (Panellist)
- *To consider context that is in favour or not* (Panellist)
- *To Create synergies depends on the context, how the context relates with different items* (Panellist)
- *Relationship is the result of context (..) you need to have the context for doing something* (Panellist)
- *It is key to understand the context including the social component of it* (Panellist)
- *Systems thinking and context overlap* (Panellist)
- *One can see context without its interconnections, systems is about seeing interconnections across all elements, context is landscape* (Panellist)

- *Entrepreneurial needs to be defined within the public health context* (Panellist)
- *Seeing the complexity of systems at work within a given context* (Panellist)
- *Context and systems thinking overlap, but you need to understand context to understand systems thinking* (Panellist)
- *You can see the context but not see the* system (Panellist)
- *Inclusion and diversity are absolutely essential for systems thinking* (Panellist)
- *Need to take a close look at the context, the way it is reflected relates to systems thinking* (Panellist)
- *Context is a pre-condition for systems-thinking* (Panellist)
- *Context determines the level to which the performance of a competencies can be realized (developed, developing country, man, women)* (Panellists)
- *Context as the overall circle with all others inside* (Panellist)
- *There is no single definition of good leadership for all and at all times, relative weight for a given leader, period of time may vary, it depends on the context in which we define leadership* (Panellist)

Self and Mind-set:
- (…) *there are overlaps between Self and Mind-set, but sometimes Self-issues are not Mind-issues* (Panellists)
- (…) *Nourishing D&I shows that you value D&I so it is about what you value out of the* self (…) (Panellist)
- (…) *Operationalisation could bring forth mind-set* (…) (Panellist)
- (…) *Self and Mind-set should be joined* (Panellist)

Process:
- (…) *you need to follow a certain process to be aligned* (…) (Panellist)
- (…) *All competencies are phrased with an active word and this refers to a process in general* (…)(Panellist)

Categories & general:
- (…) *categories are never distinctly described, they are interrelated* (…) (Panellist)
- (…) *To tease out the difference between leadership and transformational leadership* (…) (Panellist)
- (…) *global scenario as overarching issue in all competencies (climate, pandemic) to recognize the multi determinants of health that lie beyond the health sector (socio-economic, political, etc.), to work transdisciplinary* (…) (Panellist)
- *Categories form a part of the framework that is given (…) Challenges (global scenario, etc.) are part of context. Need to specify context clearly within these categories* (…) (Research group member)
- (…) *Organisation or the way the person you are supervising/leading see you as an organized person* (…) (Panellist)
- (…) *Competencies are overlapping, to think of them as a Venn* diagram (…) (Panellist)
- (…) *show framework as a gradient rather than distinct categories to capture complexities in different work places (…) one competency can fall into more than one category, perfectly okay* (Panellist)
- (…) *One needs to have integrity, some ability to comfortable with uncomfortable things, processes, etc.* (…) (Panellist)
- (…) *Need to capture human competencies like compassion, empathy, humility, resilience* (…) (Panellist)
- (…) *Include the deeper dimension of emotional intelligence, nurturing your emotional intelligence* (…)(Panellist)

Explanation for categorization

C01:
- EvaSys survey clear decision for Cat 1=Process (45.5%).
- Consensus vote resulted in a decision for Cat 1=Process

C02:
- EvaSys survey clear decision for Cat 2 =Context (54.5%).
- Consensus vote resulted in a decision for Cat 2=Context with a component on Systems Thinking.

C03:
- EvaSys survey clear decision for Cat 5= Relationships (59.1%).
- Consensus vote resulted in a decision for Cat 5=Relationship and Cat1=Process

C04:
- EvaSys survey clear decision for Cat 3 =Self (40.9%).
- Consensus vote resulted in a decision for Cat 3=Self and Cat5=Relationship

C05:
- EvaSys survey clear decision for Cat 5 =Relationship (50%).
- Consensus vote resulted in a decision for Cat5=Relationship and then combination of Cat 3=Self and Cat 6= Mind-set

C06:
- EvaSys survey decision for Cat 2 =Context (40.9%).
- Consensus vote resulted in a decision for Cat2=Context and Cat5=Relationship at the same level

C07:
- EvaSys survey decision for Cat 3 =Self (50%).
- Consensus vote resulted in a (strong) decision for Cat3=Self

C08:
- EvaSys survey decision for Cat 3 =Self (68.2%).
- Consensus vote resulted in a decision for an overlap of Cat3=Self & Cat6= Mind-set

C09:
- EvaSys survey decision for Cat 1 =Process (27.3%) and Cat 5= Relationships.
- Consensus vote resulted in a decision for a Mix of Cat5= Relationship and Cat1= Process

C10:
- EvaSys survey clear decision for Cat4 =Systems thinking (81.8%).
- Consensus vote resulted in a decision for Cat2= Context and Cat5= Relationships

C11:
- EvaSys survey clear decision for Cat5 =Relationships (40.9%).
- Consensus vote resulted in a decision that CO11 need to be revised because there are two parts that are bringing in too many components.

C12:
- EvaSys survey clear decision for Cat3 =Self (59.1%).
- Consensus vote resulted in a decision for Cat3= Self with an overlap of Cat6=Mind-set

C13:
- EvaSys survey no clear decision (Cat1=22,7%, Cat5=18.2, Cat6=27.3%)
- Consensus vote resulted in a decision for a mix of Cat1=Processes, Cat3= Self, Cat5=Relationships and Cat6=Mind-set

C14:
- EvaSys survey decision for Cat1=Process. Consensus vote resulted in a decision for Cat6=Mind-set

Adaptations after Delphi Round 4 (EvaSys Survey)
C01:<br>• Only 70% agreed with the formulation.<br>• To “adapt” considered an inappropriate term for leaders, as it would be too passive.<br>• There are more conditions for achieving shared goals than just adapting to the needs of the eco-system. Similarly, adapting to the needs of the eco-system is a condition serving more than just achieving shared goals.<br>• The adaption is also necessary for being able to implement change that sticks<br>C02:<br>• Agreement: 90%.<br>• Leaders do not commit a single goal.<br>• Leaders must juggle different goals, some intermediate and others long term.<br>• The long term goal is a composite of many goals.<br>C03:<br>• Agreement: 100%. Good as it is.<br>C04:<br>• Agreement: 100%. Good as it is.<br>C05:<br>• Agreement: 90%. Good as it is.<br><br>C06:<br>• Agreement: 90%.<br>• Not only monitoring, but also management and steering.<br>C07:<br>• Agreement: 80%. Good but reduced by one word.<br>C08:<br>• Agreement: 90%. Shortened, to act with integrity and yet not being ethically very difficult, thus just “integrity”<br>C09:<br>• Agreement: 90%. “Acts” would require a modifier (acting in a particular manner)<br><br>C10:<br>• Agreement: 77.8%. → was taken out.<br>• “Power relationships can vary based on cultural contexts. Might be too difficult to change. Better to focus energy on the doables. The distribution of power cannot be equal. Even in leadership, the leader would have more power entrusted in him or her. I think challenging unequal distribution is not beneficial. Instead, it should be fighting for just use of power.”<br><br>C11:<br>• Agreement: 80%. Combined with C12 → C10<br>C12;<br>• Agreement: 70%. Combined with C11 → C10.<br>• Entrepreneurial would be more related to economics and focusses on the present and a narrow paradigm linked with some western values (capitalism, neoliberalism).

5. Questionnaires

Figure S3 Questionnaire Round 1

MUSTER

EvaSys	[Copy]	Electric Paper

Bitte so markieren: ☐ ☒ ☐ ☐ ☐ Bitte verwenden Sie einen Kugelschreiber oder nicht zu starken Filzstift. Dieser Fragebogen wird maschinell erfasst.
Korrektur: ☐ ■ ☐ ☒ ☐ Bitte beachten Sie im Interesse einer optimalen Datenerfassung die links gegebenen Hinweise beim Ausfüllen.

Swiss TPH — Swiss Tropical and Public Health Institute; University of Basel; Maastricht University; CAPHRI

Delphi Study on Core Competencies for Transformational Leadership

Thank you for agreeing to take part in this Delphi Study.

Relevance and growth potential of competencies in different contexts
Competencies are context-bound and understood as a process of growth with mutual dependencies and interactions. The respective context (e.g. PhD programme, job position), corresponds to a gear that stimulates this process illustrated in the following figure (Anderson and Anderson, 2002). The process is divided into three phases, the educational context (namely a PhD programme in Health Sciences), the transition phase and the professional context, all based on the individual inner constitution. Competencies in the different phases may have different potential for development. Furthermore, certain competencies are the prerequisite for developing other, more complex competences. The allocation of specific competencies to each of the three phases will be the subject of the online workshop.

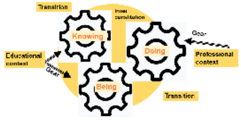

F7298U0P1PL0V0 — 23.04.2021, Seite 1/26

MUSTER

Figure S4 Questionnaire Round 2

MUSTER

evasys	DelphiStudyTL_Round2 [Copy]	evasys

Based on the feedback and data analysis of the first Delphi round, we have realigned and adjusted the list of competencies that describe transformational leaders. One fundamental change involved the categorisation of competencies into "Knowing", "Being" and "Doing" (see newsletter, Nugget 7).
The content data analysis revealed that a competency cannot be divided into these three categories. A transformational leader is rather a person who is characterised by exactly the combination of these categories (Knowing, Being and Doing) and less by the individual categories per se.

After the second round is completed we will re-analyse all the data and, based on the feedback revise the competencies again and adjust the Competency framework for the ***Online Consensus Workshop*** *on* ***19 October 2021****.*

In distinction to the first round, we ask you this time to spontaneously choose one perspective to which you feel you belong the most or which you think you have the most interfaces with regard to your current field of work:

- *Education*
- *Labour market*
- *Human resource or organisational perspective*
- *Research and Development.*
- *Policy (Adapted from Van Loo and Semeijn, 2004)*

Please answer all competency-related questions by rating to what extent you consider each competency important for ***Transformational Leadership in Public Health****. There are no right or wrong answers. It is your opinion we are interested in. You might also like to write a few words about your answer in the text box below each competency-related question.*

Please note that consent to participate is implied by completing and submitting the survey. Deadline: **September 15th 2021, 23.59 (CET/UTC+2)**.

For further questions about competency frameworks used, please contact barbara.buerkin@swisstph.ch

Please begin when you feel ready.

PART A:
Personal details

Please indicate your gender	☐ Female<br>☐ I prefer not to say	☐ Male	☐ Diverse
How old are you?	☐ Younger than 25<br>☐ 46-65	☐ 25-35<br>☐ Above 65	☐ 36-45<br>☐ I prefer not to say
What is your nationality?			
In which country is your main place of work?			
**What is the highest level of education you have achieved?**	☐ PhD degree or above<br>☐ High-school	☐ Master's degree<br>☐ Other	☐ Bachelor's degree<br>☐ I prefer not to say
Please specify:			
**Please select the perspective that best applies to your current field of work.**	☐ Education<br>☐ Research and Development<br>☐ I prefer not say	☐ Labour Market<br>☐ Policy	☐ Human Resource or Organisational Perspective<br>☐ Other

F7645U0P2PL0V0 — 30.08.2021, Seite 2/11

MUSTER

Figure S5 Questionnaire Round 3

MUSTER

evasys	DelphiStudyTL_Online Consensus Workshop	evasys

Bitte so markieren: ☐ ☒ ☐ ☐ ☐ Bitte verwenden Sie einen Kugelschreiber oder nicht zu starken Filzstift. Dieser Fragebogen wird maschinell erfasst.
Korrektur: ☐ ■ ☐ ☒ ☐ Bitte beachten Sie im Interesse einer optimalen Datenerfassung die links gegebenen Hinweise beim Ausfüllen.

Swiss TPH
Swiss Tropical and Public Health Institute
University of Basel
Maastricht University
CAPHRI

Delphi Study on Core Competencies for Transformational Leadership

Dear Expert,

Please assign each competency to __one__ of the categories that you consider to be the __most__ appropriate. Each category should appear at least once.

Cat 1 = PROCESS
Description: How and why you achieve a result or goal given a constantly changing environment. The road from A to Z.

Cat 2 = CONTEXT
Description: The ecosystem in which one operates that requires a good understanding of this system including cultural, social, economical, systemic, etc. factors

Cat 3 = »SELF»
Description: Who you are and what your value system and moral intelligence is.

Cat 4 = SYSTEMS THINKING
Description: Seeing a system as an interdependent whole and not as a collection of independent parts.

Cat 5 = RELATIONSHIPS
Description: Openness to and effective social interaction with diverse audiences across organisational levels and sectors.

Cat 6 = MIND-SET
Description: Focus based on authentic inner place allowing to drive actions based on integrity.

Adapted list of Competencies for Transformational Leadership

**C01: "*Co-creates value based, innovative solutions*".**	☐ Cat 1 = PROCESS	☐ Cat 2 = CONTEXT	☐ Cat 3 = »SELF»
	☐ Cat 4 = SYSTEMS THINKING	☐ Cat 5 = RELATIONSHIPS	☐ Cat 6 = MIND-SET
**C02: "*Adapts appropriately to the needs of the ecosystem to achieve shared goals*".**	☐ Cat 1 = PROCESS	☐ Cat 2 = CONTEXT	☐ Cat 3 = »SELF»
	☐ Cat 4 = SYSTEMS THINKING	☐ Cat 5 = RELATIONSHIPS	☐ Cat 6 = MIND-SET
**C03: "*Initiates, monitors and measures coalitions and partnerships with diverse stakeholders*".**	☐ Cat 1 = PROCESS	☐ Cat 2 = CONTEXT	☐ Cat 3 = »SELF»
	☐ Cat 4 = SYSTEMS THINKING	☐ Cat 5 = RELATIONSHIPS	☐ Cat 6 = MIND-SET

F7868U0P1PL0V0 18.10.2021, Seite 1/3

MUSTER

Figure S6 Questionnaire Round 4

MUSTER

evasys	DelphiStudyTL_Round 4	evasys

Competency 2:

"Inspires others to commit to a common goal." [Fortsetzung]

Do you agree with the formulation of this competency? ☐ Yes ☐ No

Please propose a new formulation:

Additional Comments (optional)

Competency 3:

"Empowers others to build upon their competencies and wisdom."

Do you agree with the formulation of this competency? ☐ Yes ☐ No

Please propose a new formulation:

Additional Comments (optional)

Competency 4:

"Practices participatory and inclusive leadership".

Do you agree with the formulation of this competency? ☐ Yes ☐ No

Please propose a new formulation:

Additional Comments (optional)

Competency 5:

"Creates synergies and fosters a diverse environment."

Do you agree with the formulation of this competency? ☐ Yes ☐ No

Please propose a new formulation:

Additional Comments (optional)

F8090U0P3PL0V0 10.12.2021, Seite 3/6

MUSTER

6. Terminology

In the context of this research project, we start from a holistic competency model. According to Van Loo and Semeijn (6) **competencies** are defined as "composites of individual attributes such as knowledge, skills, and attitudinal or personal aspects that represent context-bound productivity. This definition was chosen to be suitable for the context of this study as it builds on the following three assumptions: first, the competency model is based on a holistic understanding, since it is a collection of personal attributes or characteristics. Secondly, the reference to performance means that competencies are something that is actually and consistently applied, which is important for measurability in the context of competency surveys. Thirdly, competencies are always to be seen in relation to a specific context, i.e., their expression or application varies according to the given circumstances and setting.

Transformational leadership is about the assumption that everything and everyone in the universe is interconnected and part of continuous change. In this study, transformational leadership is defined as a "combination of practical skills such as modelling the way, inspiring a shared vision, challenging the process, enabling others to act and encouraging the heart to jointly bring reality to a higher level" (adapted from Kouzes & Posner (7)). Further, transformational leadership is to be seen as an individual competency development process. A stage model was designed to represent transformational leadership as a developmental process. Different stages reflect the level of experience within one competency (see Figure 3).

Competencies can be studied from different **perspectives**. Different viewpoints have different definitions and classification systems of competencies. This has implications on how competencies are measured as this is closely linked to the definitions, the leaning theories behind as well as the classifications. Van Loo and Semeijn (6) assign competencies to three different perspectives to generate a comprehensive picture on how to operationalise and describe the meaning of competencies: The educational perspective, the labour market perspective and the organisational/human resource perspective. The educational perspective argues that not only knowledge, skills and attitudes should be considered but also the learning behaviour. This perspective is therefore a rather dynamic concept. Nevertheless, performance criteria are often used today to describe competencies. The labour market perspective is traditionally very strongly oriented towards specific qualifications, which focus less on the individual and more on a specific work context with its respective and characteristic requirements. In addition, skills are very often mentioned in this area, which is traditionally used as a form of operationalizing productivity. The competency concept applied to the organisational/ human resource perspective is strongly oriented towards organisational goals. Cognitive learning as well as social learning are given importance. Cognitive learning is reflected in acquired knowledge, skills, and attitudes whereas social learning refers to agreement and satisfaction. Within the human resource perspective, competencies are directly linked to the individual, thus competencies are something dynamic rather than static. What constitutes and defines a competency is extremely contextual and subjective. The sector or professional field a person belongs to determines the type of competencies that are considered important. This does not necessarily have to be justified by the type of work performed. Rather, competencies can be weighted differently because they reflect different value patterns and experiences.

7. Defining descriptors for competencies

Box S9 Draft Operationalization of competencies (descriptors)

C01: Adapts according to the needs of the eco-system
➢ I apply principles to guide change and feel competent to initiate change processes.<br>➢ I consider alternative options and new approaches and I am always willing for negotiations.<br>➢ I'm attentive and watchful to external realities.<br>➢ I handle challenges with change.<br>➢ I'm taking the initiative to change the way things are<br>➢ I give others the chance to create new approaches and take the initiative.<br>➢ I constantly analyse the changing nature, key factors and resources that shape global health<br>(8) (7)
C02: Inspires others to commit to common goals.
➢ I apply tools and techniques to develop a common strategy.<br>➢ I initiate and structure goal-setting processes.<br>➢ I regularly use techniques to identify and pursue common goals over the long-term.<br>➢ I prospect and describe an image of the future to engage the minds and hearts of others and to anticipate what is possible.<br>➢ I trust my abilities to make extraordinary things happen.<br>➢ I clearly envision and belief in the goals, outcome, etc. before starting any project.<br>➢ I connect my visions and the future to experiences to understand the themes that led me to where I am.<br>➢ I engage others in a dialogue about the future by showing them how they will be served in the long-term.<br>➢ I generatively listen to my constituents (their hopes, dreams, and aspirations to be aware of what is meaningful and valuable to them and to identify opportunities for them).<br>(7) (9) (2)
C03 Empowers others to build upon their competencies and wisdom.
➢ I support my constituents in dealing with change and I am aware of their need for security and steadiness.<br>➢ I express my trust and genuine belief in the capacity of my constituents to build their confidence in their abilities and their trust in me.<br>➢ I ensure the availability of professional development opportunities for the organization (e.g., training, mentoring, peer advising, coaching).<br>➢ I consult the opinions and ideas of my constituents to update and question my own perspective and offer a chance to do things differently.

- I coach my constituents step-by-step including constructive feedback, probing questions and active teaching to promote gradual progress.
- I assess my constituents' capacities and try to liberate their visions to perform in the context of the challenges they face.
- I sense my constituents' development needs in a given position and plan learning to bolster their abilities.
- I make it safe for others to try, fail, and learn from experiences and turn them into learning experiments.
- I facilitate a "can-do" attitude and increase self-determination by creating opportunities for people to accomplish a task.
- I motivate performance by supporting meaningful assignments and by creating possibilities to exercise choice and latitude.
- I assist others in positioning themselves and stating why they do what they do and what is important to them.

(1, 5) (10)

C04 Practices participatory and inclusive leadership.

- I foster accountability and responsibility by delegating authority, sharing power and involving my constituents in decisions.
- I create a spirit of community by acknowledging values and victories and by celebrating individual and team accomplishments.
- I'm striving try to build trust and psychological safety by paying attention to my tone of voice, posture, gestures, and facial expressions.
- I give my constituents the chance to explore better ways of doing things and to move forward and embrace the initiative.
- I provide challenging assignments together with support and sufficient resources to accomplish them.
- I promote group morale and productivity by providing clear directions and priorities to my constituents.
- I instruct, coach and guide others to harmonise their actions with the shared values.
- I establish challenging targets for my constituents' scope of responsibility.

(5) (11)

C05 Creates synergies and fosters a collaborative environment.

- I recognise achievements by giving personal, precise and visible feedback.
- I regularly meet with small groups and "walk the halls and plant floors".
- I sense others' feelings and perspectives, and taking an active interest in their concerns.
- I organise teamwork and coordinate interdisciplinary teams to synergise complementary skills.
- I identify and respond to underlying attitudes or behaviour patterns such as cultural norms and personality differences.
- I actively foster a diverse and tolerant environment that values singularities as well as common grounds.
- I value the diversity of my constituencies and at the same time, I build on the establishment of shared values.
- I communicate the shared values of my constituents and enable them to talk about their values with others.
- I facilitate the discussion and resolution of conflicts or disagreement.
- I analyse my environment for information enabling me to develop better communication strategies.
- I purposefully react to frustrations trying to negotiate and resolve disagreements.
- I promote and participate in socially and culturally sensitive multidisciplinary teams.

Feedback EvaSys, (5, 8) (1)

C06 Proactively manages partnerships with diverse stakeholders.

- I constantly get people and organizations interacting towards cooperative goals and promote a sense of mutual dependence and respect.
- I am looking for opportunities for interdisciplinary and intersectoral exchange among stakeholders and across continents.
- I set up and utilise networks, multi-stakeholder meetings to seek information of strategic importance.
- I conduct, coordinate, mediate and/or moderate high-level fora and events, at national, sub regional or regional levels.
- I establish formal agreements (e.g., MOUs, bilateral agreements) at various levels and promote transnational, intersectoral initiatives to overcome inequities, implement effective interventions and monitor the success of collaborations.
- I advise stakeholders and give feedback in terms of ideas, concerns or problems they are facing.
- I improve communication processes by building trust and psychological safety.
- I make an effort to hear and respond to a group's emotional currents and power relationships and anticipate other's reactions.
- I adapt my negotiation style to the respective context (e.g., competing, cooperative, avoiding, compromise, accommodating).
- I communicate complex issues clearly and credibly with widely varied audiences.
- I attempt to understand the values of others and to establish values that can be shared by all.
- (5, 8) (1) (10) (11)

C07 Personifies optimism and perseverance to build resilience in challenging times.

- I ask for clarification if I feel that I have not understood something.
- I consider problems as constructions dependent on time and situation that arise due to its problematic description, explanation and assessment.
- I operate with the confidence that behaviour is situational and contextual and therefore not constant.
- I value having others question or challenge my approaches.
- I see challenges and disappointments as learning opportunities to generate small wins and am curious about the uncertainties that life holds.
- I constantly try to demonstrate a positive and engaging behaviour seeing the positive aspects of things and the future.
- I have an awareness of my emotions and apply techniques to balance disturbing emotions and impulses.
- I embrace opportunities for learning and constantly become involved and engaged to what's happening.
- I preserve an objective attitude within negotiations and a non-emotional distance from interpersonal conflicts.

(5) (1)

C08 Aspires to act with integrity.

- I openly disclose things I stand for, I value, I want, I hope for and I am willing (not willing) to do and thus give up control and show vulnerability.
- I apply self-regulation techniques to operate authentically.
- I continuously try to personify a connection between action and the values I stand for.
- I am aware of my core values and guiding principles and beliefs by examining experiences to make choices and decisions.

- I formulate the values that guide my decisions, priorities and actions and I am mindful of my choice of words.
- I strive to improve my emotional intelligence (through training, mentoring and feedback from others).
- I regularly reflect my own beliefs, values and behaviours and the ways in which these can impact both my decision making and the response to others.
- I clarify my vision (What drives me? What do I care about? Why do I want to accomplish certain things?)
- I attempt to give constructive and precise feedback based on facts and behavioural patterns observed, containing individualised suggestions for improvement.
- I get personally involved and demonstrate with my behavior what is desired, expected and what will be rewarded (e.g., by attending meetings and events, visit stakeholders, make presentations, visiting my constituents at their desks to say hello).
- I demonstrate cultural sensitivity and incorporate ethical standards of practice (e.g., Public Health Code of Ethics) into all interactions.
- I use ethical principles to critically review the development and implementation of policies and activities.

(8) (5) (11) (10) (11) Feedback EvaSys Round 4

C09 Acts effectively in complex and multifaceted systems.

- I engage in change initiatives at a micro (individual), meso (group), macro (institutional) as well as the global level.
- Encourages others to see the positive outcome of doing things differently.
- I try to make connections across seemingly unrelated questions, problems or ideas.
- I promote a comprehensive, strategic perspective and at the same time focus on the crucial details.
- I use a variety of analyses and experiences to draw conclusions.
- I use my depth-expertise in how to facilitate and manage change at a micro (individual), meso (group), macro (institutional) as well as the global level.
- I actively seek and identify emerging development opportunities and take the initiative to change the way things are done.
- I experiment with many ideas and divide larger problems into small, manageable actions.
- I'm willing to do things that have never been done before and actively put myself in new situations.
- I continuously change my perspective to assess and promote global responsibility in terms of local actions as well as to understand the local impact of global events.
- I reprioritise and restructure regularly to increase my constituents' responsiveness to internal and external demands.
- I try to overcome habitual patterns of thinking and welcome opportunities to step out of my comfort zone/ by actively looking for new ways of doing things and by perceiving themes or patterns in seemingly random items, events, or phenomena.

(5, 8) (1)

C10 Pursues strategic approaches for value-based, innovative solutions.

- I synthesize information and ideas from multiple sources and disciplines to design and implement of programs, policies, and systems.(11)
- I assess cultural, ethical, environmental, and political influences on the feasibility and sustainability of activities.
- I actively involve my constituents in a dialogue/discussion about values and make them reflect on core values.
- I ensure that people comply with agreed values and norms.
- I evaluate the performance and impact of actions and strategies.
- I assess risks, benefits and unforeseen consequences of our professional work.
- I direct organisational decision-making and planning based on internal and external research.
- I develop professional plans that include lifelong learning, mentoring and strategies for continuous professional development
- I apply quality improvement methods.
- I attempt to stay up to date with changing trends and stay sensitive to changing external circumstances.

(5, 7) (11)

References Competency Frameworks

1. Boyatzis R, Boyatzis RE. Competencies as a behavioral approach to emotional intelligence. Journal of Management Development. 2009.
2. WHO. WHO-ASPHER Competency Framework for the Public Health Workforce in the European Region. WHO Regional Office for Europe; 2020.
3. Calhoun JG, McElligott JE, Weist EM, Raczynski JM. Core competencies for doctoral education in public health. American journal of public health. 2012;102(1):22-9.
4. Anderson D, Anderson LA. Beyond change management: Advanced strategies for today's transformational leaders: John Wiley & Sons; 2002.
5. OECD. Competency Framework. Paris2014.
6. Van Loo J, Semeijn J. Defining and measuring competences: an application to graduate surveys. Quality & Quantity. 2004;38(3):331-49.
7. Kouzes JM, Posner, Barry Z. The leadership challenge: How to Make Extraordinary Things Happen in Organizations. 5th edition. ed. Posner BZ, editor. San Francisco: Jossey-Bass; 2012.
8. World Health Organization. Core competencies for public health: a regional framework for the Americas. Washington (DC): Pan American Health Organization2013.
9. Scharmer O. The essentials of theory U: Core principles and applications: Berrett-Koehler Publishers; 2018.
10. Public Health Foundation. Council on Linkages Between Academia and Public Health Practice. Core Competencies for public health professionals. 2014.
11. Association of Schools of Public Health Education Committee. Doctor of public health (DrPH) core competency model, version 1.3. Washington: ASPH. 2009.

8. Self-assessment tool: Competencies for Transformational Leadership

Core Competencies for Transformational Leadership
Self-assessment tool

This self-evaluation tool is the result of five rounds of an expert informed Delphi process. The Competency Framework including competencies, categories and descriptors were systematically developed using quantitative and qualitative analytical methods.

The self-assessment tool is designed to support you in reflecting on your personal development status. It should also help you to identify areas for further development.

Procedure:

1. *Read the competency and its descriptors.*
2. *Next to each descriptor, you will find a scale, please choose a rating that best reflects how often this behaviour applies to you.*
3. *Now add up the values in each column and write the value at the bottom of the competency.*
4. *Reflect on how you have rated yourself. If you have mostly light green scores within a competency area, this could be a competency you would like to develop further. If you have almost exclusively dark green ratings, this could be a sign that certain strengths and skills are being overused. It could be a behaviour that you rely on too much, which could have a negative impact on your overall performance.*

**Competency 1**	**Adapts according to the needs of the eco-system**			
	Very little	Some of the time	Many times	Always
1. I apply principles to guide change and feel competent to initiate change processes.				
2. I constantly analyse the changing nature, key factors and resources that shape global health.				
3. I'm attentive and watchful to external realities.				
4. I consider alternative options and new approaches and I am always willing for negotiations.				
5. I handle challenges with change and flexibility.				
6. I recognise new aspects and align my observations on the basis of experience, which enables me to grasp processes easily.				
7. I give others the chance to create new approaches and take the initiative.				
8. I immerse myself in the context and try to get the best out of it.				
9. I feel comfortable to step back in full confidence that my team will continue in an autonomous, target-driven and situational way.				
**TOTAL**				

**Competency 2**	**Inspires others to commit to common goals**			
	Very little	Some of the time	Many times	Always
1. I regularly apply participative leadership techniques (e.g., Art of Hosting, Transformational scenario planning, Experimentation) with my team and/or external partners.				
2. I initiate and structure goal-setting processes.				
3. I clearly envision the goals, outcome, etc. before starting any project.				
4. I actively participate in the career planning and development of my team to ensure that personal goals are clearly defined.				
5. I ensure that goals are understood collectively.				
6. I connect my visions and the future to personal experiences to understand the themes that led me to where I am.				
7. I try to empathise with the reasons for different behaviours of my employees.				
8. I project an image of the future to inspire others to envision what is possible and how their own interests will be served at the same time.				
9. I generatively listen to my constituents (their hopes, dreams, and aspirations to be aware of what is				

meaningful and valuable to them and to make them aware of opportunities).				
**TOTAL**				

**Competency 3**	**Empowers others to fully capture and build upon their competencies and wisdom**			
	Very little	Some of the time	Many times	Always
1. I ensure the availability of professional development opportunities for the organization (e.g., training, mentoring, peer advising, coaching).				
2. I facilitate a "can-do" attitude and increase self-determination by creating opportunities for people to accomplish a task.				
3. I motivate performance by giving meaningful assignments and by creating possibilities to exercise choice and latitude.				
4. I assess my constituents' capacities and try to liberate their visions to perform in the context of the challenges they face.				
5. I consult the opinions and ideas of my constituents to update and question my own perspective and offer a chance to do things differently.				
6. I support my constituents in implementing their ideas even if it does not fully align with the way I would do things.				
7. I support my constituents in dealing with change and I am aware of their need for security and steadiness.				
8. I support others to be authentic in the way they act (e.g., positioning themselves, stating what is important to them).				
9. I encourage my constituents to build their confidence in their abilities and their trust in me.				
10. I make it safe for others to try, fail, and learn from experiences.				
11. I coach my constituents step-by-step including constructive feedback, probing questions and active teaching to promote gradual progress.				
**TOTAL**				

**Competency 4**	**Practices participatory and inclusive leadership**			
	Very little	Some of the time	Many times	Always
1.I appreciate to work with people of different backgrounds including socio-economic status, different nationalities, different sex/gender, etc.				
2. I invite constituents to co-create work processes and projects, from ideation to evaluation of the implementation.				
3. I promote group morale and productivity by providing clear directions and priorities to my constituents.				
4. I provide challenging assignments and targets for my constituent's scope of responsibility together with support and sufficient resources to accomplish them.				
5. I create a spirit of community by acknowledging values and victories and by celebrating individual and team accomplishments.				
6. I give my constituents the chance to explore better ways of doing things and to move forward and embrace the initiative.				
7. I foster accountability and responsibility by delegating authority, sharing power and involving my constituents in decisions.				
8. I am striving to build trust and psychological safety by paying attention to my tone of voice, posture, gestures, and facial expressions.				
9. I feel comfortable working with people that do not share my own values to try and build a consensus.				

I strive to ensure that the structure of my team, as well as our activities, reflect the composition and needs of the communities we aim to serve.				
**TOTAL**				

**Competency 5**	**Creates synergies and fosters a collaborative environment**			
	Very little	Some of the time	Many times	Always
1. I recognise achievements by giving personal, precise and supportive feedback.				
2. I sense others' feelings and perspectives, and take an active interest in their concerns.				
3. I identify and respond to underlying attitudes or behaviour patterns (e.g. cultural norms, personal preferences and differences).				
4. I support my constituents in dealing with change and I am aware of their need for security and steadiness.				
5. I facilitate the discussion and resolution of conflicts or disagreement.				
6. I analyse my environment for information enabling me to develop better communication strategies.				
7. I purposefully react to frustrations trying to negotiate and resolve disagreements.				
8. I regularly walk the halls and workspaces to chat and have informal conversations.				
9. I actively foster a diverse and tolerant environment that values singularities as well as common grounds.				
10. I value the diversity of my constituencies and at the same time, I build on the establishment of shared values.				
11. I communicate the shared values of my constituents and enable them to talk about their values with others.				
12. I organise teamwork and coordinate interdisciplinary teams to synergise complementary skills.				
13. I promote and participate in socially and culturally sensitive multidisciplinary teams.				
14. I encourage academia and/or international agencies to take the lead in organizing multisectoral workshops (public & private sectors, civil society, bilateral agencies, donors) to address any given multifactorial problem (e.g. health, environment, poverty) analyse potential risk factors and underlying determinants and develop jointly a methodology using e.g. logframe to define process and impact indicators which cut across sectors. Publish the findings.				
15. I encourage utilization of the workshop findings and recommendations to move them forward (e.g pilot projects).				
**TOTAL**				

**Competency 6**	**Proactively manages partnerships with diverse actors**			
	Very little	Some of the time	Many times	Always
1. I attempt to understand the values of others and to identify values that can be shared by all.				
2. I explore any potential conflict of interests with any given partner before setting up a partnership.				
3. I research and listen to each of the partners interests.				
4. I engage partners in every step of the process, initially in informal settings to build trust and camaraderie before moving on to settings that are more formal.				

5. I advise constituents and give feedback in terms of ideas, concerns or problems they are facing.				
6. I try to communicate complex issues clearly and credibly with widely varied audiences.				
7. I try to be fully aware of any possible cultural sensitivities that might jeopardize the partnership and prepare to handle these.				
8. I constantly try to encourage people and organizations interacting towards cooperative goals and promote a sense of mutual dependence and respect.				
9. I make an effort to hear and respond to a group's emotional currents and power relationships and anticipate other's reactions.				
10. I adapt my negotiation style to the respective context (e.g. competing, cooperative, avoiding, compromise, accommodating).				
11. I am looking for opportunities for interdisciplinary and intersectoral exchange among actors and across continents.				
12. I conduct, coordinate, mediate and/or moderate high-level fora and events, at national, sub regional or regional levels.				
13. I establish formal agreements (e.g. MOUs, bilateral agreements) at various levels and promote transnational, intersectoral initiatives to overcome inequities, implement effective interventions and monitor the success of collaborations.				
14. I encourage consensus building to decide on the best possible partnership's management body/entity (with special care on conflict of interest issue).				
**TOTAL**				

**Competency 7**	**Personifies optimism to build resilience and perseverance in challenging times**			
	Very little	Some of the time	Many times	Always
1. I constantly try to demonstrate a positive attitude by practicing a solution-oriented approach.				
2. I embrace opportunities for learning and constantly become involved and engaged to what is happening.				
3. I approach problems as potential for growth and scope for experience in a specific context.				
4. I see challenges and disappointments as learning opportunities to generate small wins.				
5. I am curious about the uncertainties that life holds.				
6. I have an awareness of my emotions and apply techniques to balance disturbing emotions and impulses.				
7. I perceive the emotions of others and then reflect on how and whether I will respond to them.				
8. I value having others question or challenge my approaches.				
9. I preserve an objective attitude within negotiations and a non-emotional distance from interpersonal conflicts.				
10. I feel competent to assess the behaviour of my team in relation to the context and the situation.				
**TOTAL**				

**Competency 8**	**Acts with integrity**			
	Very little	Some of the time	Many times	Always
1. I ask for clarification if I feel that I have not understood something.				
2. I adhere to honest and verifiable research methods in connection with research projects (proposal, implementation and evaluation) and follow generally accepted scientific norms and standards. (New)				
3. I communicate research results with a special focus on adherence to rules, regulations, and guidelines.				

4. I formulate the values that guide my decisions, priorities and actions and I am mindful of my choice of words.				
5. I apply self-regulation techniques (e.g. meditation, distraction, imagination, physical activity) to ensure that I operate in an authentic way.				
6. I am aware of my core values and guiding principles and beliefs by examining experiences to make choices and decisions.				
7. I attempt to give constructive and precise feedback based on facts and behavioural patterns observed, containing individualised suggestions for improvement.				
8. I openly disclose things I stand for, I value, I want, I hope for and I am willing (not willing) to do.				
9. I continuously try to personify a connection between action and the values I stand for.				
10. I demonstrate cultural sensitivity and incorporate context specific ethical principles (e.g., Public Health Code of Ethics) into all interactions (including the development and implementation of policies and activities).				
11. I get personally involved and demonstrate with my behavior what is desired, expected and what will be rewarded (e.g. by attending meetings and events, visit actors, make presentations, visiting my constituents at their desks to see how they are, how they are feeling, to listen, to give support, etc.				
12. I strive to improve my emotional intelligence (through training, mentoring and feedback from others).				
13. I regularly reflect my own beliefs, values and behaviours and the ways in which these can impact both my decision making and the response to others (What drives me? What do I care about? Why do I want to accomplish certain things?)				
**TOTAL**				

**Competency 9**	**Operates effectively within and across complex and multifaceted systems**			
	Very little	Some of the time	Many times	Always
1. I (pro-)actively facilitate and engage in change initiatives at a micro (individual), meso (group), macro (institutional) as well as the global level.				
2. I encourage others to see the positive outcome of doing things differently.				
3. I promote a comprehensive, strategic perspective and at the same time focus on the crucial details.				
4. I use a variety of analyses and experiences to draw conclusions.				
5. I actively seek and identify emerging development opportunities and take the initiative to change the way things are done.				
6. I experiment with many ideas and divide larger problems into small, manageable actions.				
7. I am willing to do things that have never been done before and actively put myself in new situations.				
8. I continuously change my perspective to assess and promote global responsibility in terms of local actions as well as to understand the local impact of global events.				
9. I reprioritise and restructure regularly to increase my constituents' responsiveness to internal and external demands.				
10. I try to overcome habitual patterns of thinking and welcome opportunities to step out of my comfort zone/ by actively looking for new ways of doing things and by perceiving themes or patterns in seemingly random items, events, or phenomena.				

11. I encourage other to make connections across seemingly unrelated questions, problems or ideas to develop comprehensive solutions.				
**TOTAL**				

**Competency 10**	**Pursues strategic approaches for value-based, innovative solutions**			
	Very little	Some of the time	Many times	Always
1. I synthesize information and ideas from multiple sources and disciplines to design and implement of programs, policies, and systems.				
2. I assess cultural, ethical, environmental, and political influences on the feasibility and sustainability of activities.				
3. I actively involve my constituents in a dialogue/discussion about values and encourage them to reflect on core values.				
4. I encourage others to comply with agreed values and norms.				
5. I evaluate the performance and impact of actions and strategies.				
6. I assess and balance risks, benefits and unforeseen consequences of our professional work.				
7. I direct organisational decision-making and planning based on shared and transparent criteria.				
8. I develop professional plans that include lifelong learning, mentoring and strategies for continuous professional development.				
9. I apply quality improvement methods including process, product and people-based improvements (New).				
10. I attempt to stay up to date with changing trends and stay sensitive to changing external circumstances.				
**TOTAL**				

9. Personal development plan

(1) *Choose one development need that you have identified for yourself (you can repeat the exercise after the course for other areas). Summarise the most important aspects of this in key points* ***(WHAT).***
(2) *Justify your choice* ***(WHY).***
(3) *Start at the end to describe the specific activities. In other words, what is the overall goal or desired new behaviour, what will be in place, what will you have achieved at the end. Think about all the activities that are necessary for you to perform this behaviour. As a tip, the activities can be broadly categorised as experience, engagement and education* ***(HOW).***
(4) *Take the 'Personal Strengths and Resources' worksheet that you have already completed in preparation. Think about which personal resources and strengths are particularly useful for your undertaking* ***(WHICH).***
(5) *In addition, think about the timing* ***(WHEN),*** *the location* ***(WHERE),*** *and possible stakeholders/groups of people* ***(WHO)*** *in your environment who can be both supportive and impeding.*

1. ***What*** **will be done? (Development need)**

2. ***Why*** **is this important (Reasons for choosing)**

3. ***How*** **will this be implemented? (Description of the activities, measures, etc.)**

4. ***Which*** **(personal) resources and strengths are useful for your undertaking?**

5. ***When*** **should this happen? (Time/sequence)**

6. ***Which*** **(personal) resources and strengths are useful for your undertaking?**

7. ***Where*** **will this be done? (Location)**

8. ***A) Who can be supportive? Who needs to be involved? (Stakeholders/persons)***

**9.** ***B) Who could impede the activity? (Stakeholders/persons)***

10. Additional information

Table S4 Rating on the importance of the competencies for transformational leadership in the first online survey

Competency	MW (mean)	MD (mode)	S (standard deviation)	Number=43	
**C01_TL**	6.3	6	0.7	43	
**C02_TL**	6.6	7	0.5	43	
**C03_TL**		7	1.0	41	
**C04_TL**	6.2	6	0.9	43	
**C05_TL**	5.8	5	1.2	41	
**C06_TL**	5.8	5	0.9	43	
**C07_TL**	5.7	6	1.0	42	
**C08_TL**	6.1	6	1.1	43	
**C09_TL**	6.4	7	0.7	43	
**C10_TL**	6.7	7	0.5	43	
**C11_TL**	6.7	7	0.7	43	
**C12_TL**	6.3	7	1.0	43	
**C13_TL**	6.4	7	0.8	43	
**C14_TL**	6.7	7	0.6	43	
**C15_TL**	6.2	6	0.9	43	
**C16_TL**	6.6	7	0.7	43	
**C17_TL**	6.3	7	0.8	43	
**C18_TL**	6.4	7	0.8	43	
**C19_TL**	5.4	6	1.3	42	
**C20_TL**	5.3	6	1.5	42	
**C21_TL**	6.3	7	0.9	43	
**C22_TL**	5.9	6	1.0	42	
**C23_TL**	6.7	7	0.7	43	
**C24_TL**	6	6	0.8	43	
**C25_TL**	6	6	1.1	42	
**C26_TL**	6.6	7	0.7	43	
**C27_TL**	6.5	7	0.7	43	
**C28_TL**	6.5	7	0.8	43	
**C29_TL**	6.4	7	0.7	43	
**C30_TL**	6.4	7	0.8	42	
**C31_TL**	6.4	7	1.2	42	
**C32_TL**	6.4	7	1.0	43	
**C33_TL**	6	6	1.2	43	
**C34_TL**	6.8	7	0.5	43	
**C35_TL**	6.1	6	1.1	43	
**C36_TL**	6.3	7	1.0	43	
**C37_TL**	6.5	7	0.8	43	
**C38_TL**	6.2	6	0.9	43	

Box S9 – Definitions superordinate competency categories

Category 1 = PROCESS
How and why you achieve a result or goal given a constantly changing environment.
The road from A to Z.

Category 2 = SELF/MIND-SET
Who you are and what your value system and moral intelligence is. Focus based on authentic inner place allowing to drive actions based on integrity.

Category 3 = SYSTEMS THINKING
Seeing a system as an interdependent whole and not as collection of independent parts.

Category 4 = RELATIONSHIPS
Openness to and effective social interaction with diverse audiences across organisational levels and sectors.

Table S5 Selection criteria for participation in the Delphi Study

**Inclusion criteria**	**Exclusion criteria**
Classification into one or more of the categories (educational perspective, labour market perspective, organisational/human resource perspective)	Refusal to participate in the Delphi study
Motivation and willingness to participate as well as to reconsider previous assessments in successive rounds	
Professional backgrounds and in-depth experiences linked to the topic area	No response after a second reminder e-mail following the preliminary invitation
